# Non-equilibrium plasmon liquid in a Josephson junction chain

**DOI:** 10.1126/sciadv.ady7222

**Published:** 2026-02-13

**Authors:** Anton V. Bubis, Lucia Vigliotti, Maksym Serbyn, Andrew P. Higginbotham

**Affiliations:** ^1^ Institute of Science and Technology Austria, Am Campus 1, Klosterneuburg 3400, Austria.; ^2^ James Franck Institute and Department of Physics, University of Chicago, 929 E 57th St., Chicago, IL 60637, USA.

## Abstract

Equilibrium quantum systems are often described by a gas of weakly interacting normal modes. Bringing such systems far from equilibrium, however, can drastically enhance mode-to-mode interactions. Understanding the resulting liquid is a fundamental question for quantum statistical mechanics and a practical question for engineering driven quantum devices. To tackle this question, we probe the non-equilibrium kinetics of one-dimensional plasmons in a long chain of Josephson junctions. We introduce multimode spectroscopy to controllably study the departure from equilibrium, witnessing the evolution from pairwise coupling between plasma modes at weak driving to dramatic, high-order, cascaded couplings at strong driving. Scaling to many-mode drives, we stimulate interactions between hundreds of modes, resulting in near-continuum internal dynamics. Imaging the resulting non-equilibrium plasmon populations, we then resolve the nonlocal redistribution of energy in the response to a weak perturbation—an explicit verification of the emergence of a strongly interacting, non-equilibrium liquid of plasmons.

## Teaser

Researchers have observed the non-equilibrium, liquid-like behavior of photons in a strongly driven superconducting circuit.

## INTRODUCTION

In wave systems, nonlinearities cause the transfer of energy and momentum between resonant modes. Long of interest in studies of solids (*1*), classical thermalization (*2*), and wave turbulence (*3*), coupled multimode dynamics has more recently gained attention in engineered quantum systems due to the development of high-quality multimode resonators in several experimental platforms. The applications of multimode resonators in quantum science are diverse, including non-Hermitian mechanics (*4*, *5*), signal processing (*6*, *7*), memory (*8*–*10*), quantum thermalization (*11*), and many-body extensions of cavity quantum electrodynamics (*12*–*14*).

Long chains of Josephson junctions are a particularly pristine realization of a one-dimensional multimode quantum system. The Josephson inductance results in a low speed of light, allowing the realization of chip-scale microwave multimode resonators. Josephson chains have long been studied as realizations of quantum *XY* models (*15*–*18*), undergoing a superconductor-insulator quantum phase transition due to a competition of Josephson and charging energies (*19*–*21*). In the microwave domain, Josephson chains are useful for quantum-limited amplification (*22*–*24*) and superinductance (*25*, *26*).

More recently, microwave spectroscopy of long chains has been used as a means to understand both superconductor-insulator (*27*, *28*) and impurity problems (*29*–*31*). Despite this progress, the frontier of high energy-density, where nonlinearities result in strong mode-to-mode interactions and the emergence of a plasmon liquid, remains open. Here and throughout, we use the term plasmon liquid in the context of nonlinear optics and wave physics, to denote strongly interacting, collective dynamics of bosonic excitations rather than the presence of a quadratic (massive) dispersion. In our system, the plasmons retain a gapless dispersion without a mass, similar to the hydrodynamic regimes of light and surface plasmons described in (*32*), rather than the massive polariton fluids found in confined cavities (*33*). What are the key internal dynamical processes of this plasmon liquid, and how can they be cleanly observed and characterized?

**Fig. 1.**
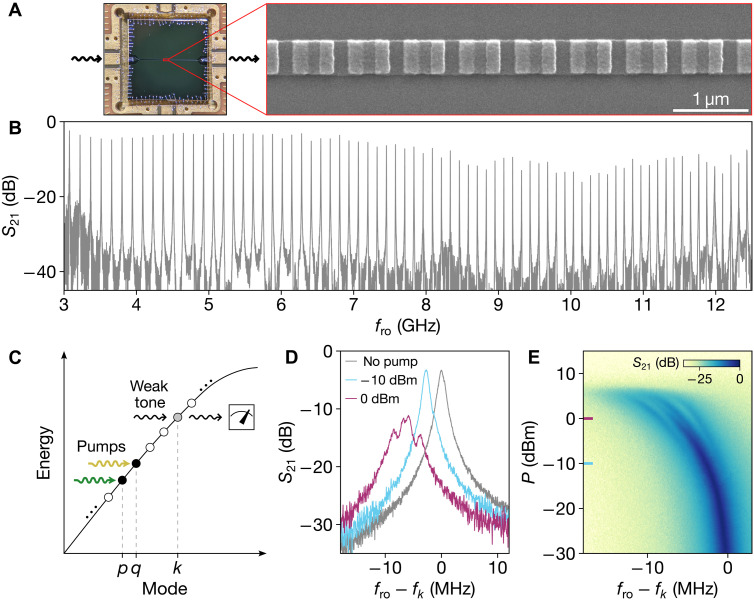
Microwave spectroscopy of the JJ-chain. (**A**) An optical micrograph of the device mounted on a sample holder, with a silicon chip measuring 7 by 7 mm. To the right, a scanning electron microscopy image of the JJ-chain is shown. (**B**) Linear response transmission magnitude ($S_{21}$) of the device with background cross-talk subtracted (see fig. S4). (**C**) Schematic of the three-tone measurement: Two coherent tones pump modes p and q, while a weak readout tone near mode k is used to measure $S_{21}$. (**D** and **E**) $S_{21}$ data measured around mode k=29 with modes p=25 and q=26 pumped at equal power P at room temperature. In (D), $S_{21}$ without pumps is plotted in gray for comparison.

Here, we address these questions by implementing multimode microwave spectroscopy to investigate the internal dynamics of Josephson junction chains. The discrete set of plasma mode excitations in the Josephson junction chains is described by an effective bosonic Hubbard model

$$H=H_{0}+H_{\text{int}}=\sum_{k>0}\hbar \omega_{k}{\hat{a}}_{k}^{†}{\hat{a}}_{k}+\sum_{\text{klmn}}\hbar K_{\text{klmn}}{\hat{a}}_{k}^{†}{\hat{a}}_{l}^{†}{\hat{a}}_{m}{\hat{a}}_{n}$$ (1)

where ${\hat{a}}_{k}^{†}$ and ${\hat{a}}_{k}$ are creation and annihilation operators of given plasma mode k. The first term $H_{0}$ captures the energies of individual modes (see Fig. 1), corresponding to the excitation of individual standing waves that are only weakly interacting in equilibrium. The second interaction term, $H_{\text{int}}$, correspond to momentum and energy-conserving multimode scattering processes described by matrix element $K_{\text{klmn}}$.

The Hamiltonian (*1*) provides a generic description of nonlinear multimode cavity and is relevant to a broad range of physical systems. Previous work in Josephson systems has focused on parametrically pumping isolated terms in $H_{\text{int}}$, including two-mode squeezing (*7*, *22*–*24*, *34*, *35*) and beam-splitter interactions (*36*). In contrast, this work seeks to understand the driven dynamics in the many-body setting, where a large number of terms in $H_{\text{int}}$ are resonant and compete with wave dispersion. Another interesting point of comparison is one-dimensional electronic systems (*37*, *38*), where nonlinear Luttinger plasmons described by Eq. 1 have been observed in several systems (*39*–*41*). From the Luttinger liquid perspective, our work opens avenues by directly measuring the predicted quasiparticle lifetime of plasmons (*32*) in an engineered superconducting circuit. From the superconducting circuit perspective, the quasiparticle lifetime of plasmons is a many-body extension of the known nonlinear damping phenomena in a nonlinear Kerr oscillator (*22*, *42*). We emphasize that the effects mentioned above, including the internal plasmon lifetime that we observe, can be largely understood from the perspective of classical nonlinear physics.

The first question we address is the origin of the nonlinearity in Eq. 1, as it is a priori unclear whether it will be dominated by the typical gradient expansion of the Josephson potential or rather by quantum phase slips—events where the superconducting phase tunnels by 2π (*43*). By weakly driving individual nonlinear terms, we determine that the matrix elements in Eq. 1 increase with wave number, signaling that quantum phase slips are negligible in the weak-driving regime. Further increasing the drive strength, we observe key features of energy and momentum redistribution in nonlinear, one-dimensional quantum systems: Cascaded couplings between modes compete with wave dispersion, resulting in strongly hybridized clusters of plasma modes. High mode occupations created by driving result in a continuous broadening of the plasma modes due to momentum-conserving scattering, which we quantify by extending the kinetic framework for thermal transport in one-dimensional quantum liquids (*11*, *32*, *38*, *43*). For the strongest drives, we observe unexpectedly large decay rates of low wave-number modes, hinting at the emergence of phase slips in a strongly non-equilibrium regime. Our findings establish Josephson junction chains as a versatile platform for investigating multimode quantum dynamics, with broader implications for one-dimensional quantum systems and circuit quantum electrodynamics.

## RESULTS

### Model system and multimode spectroscopy

We study a 5-mm-long chain of Josephson junctions as a model for a non-equilibrium quantum many-body system. The sample is fabricated using standard electron-beam lithography and double-angle shadow evaporation of Al/AlO*_x_*/Al junctions (Fig. 1A). Both ends of the chain are connected to microwave lines within a dilution refrigerator, enabling measurements of transmission and reflection (see setup details in fig. S1). All measurements are performed at a base temperature of 12 mK. The linear response transmission, $S_{21}$, as a function of the readout frequency $f_{\text{ro}}$, reveals a dense series of transmission peaks at frequencies $f_{k}$ (Fig. 1B), corresponding to the plasma modes of the chain in Eq. 1 (*26*), $f_{k}\approx \omega_{k}/\left(2\pi \right)$, where the deviation stems from the self-energy correction due to Kerr effect (*44*, *45*). Although the chain is galvanically coupled to the microwave launchers, boundary conditions defining discrete set of modes arise naturally due to the impedance mismatch between the 50 Ω network and the chain, which has a high characteristic impedance of *Z* > 10 kΩ (see table S1), typical for superinductors (*27*). Plasma modes are labeled by an integer number k=1,… and can be thought as counterpropagating standing waves with quasimomenta ∝±k. Consequently, two mode scattering processes m,n→k,l in Eq. 1 conserve momenta modulo sign, ∣m∣+∣n∣=∣k∣+∣l∣.

A first glimpse of the response to multimode drives is given by exciting two modes, p and q (here and below p<q) with pump tones, while measuring a third mode, k, with a weak, linear response readout tone (Fig. 1C). For low pump-tone power, the readout mode experiences a Kerr-like frequency shift arising from the self-energy correction terms in $H_{\text{int}}$ (see Eq. 1), while the transmission peak retains its Lorentzian shape (*26*, *44*, *46*). At elevated pump powers, the transmission peak splits, with up to four distinct peaks resolved (Fig. 1D). This splitting smoothly emerges with increasing pump power P (see Fig. 1E). Below, we show that the observed non-Lorentzian peak shape originates from the interaction term in Eq. 1, highlighting the fragility of the individual modes with respect to intermode interactions. By tuning the driving, we explore the effect of interactions and the fate of modes under weak, strong, and many-mode driving conditions.

**Fig. 2.**
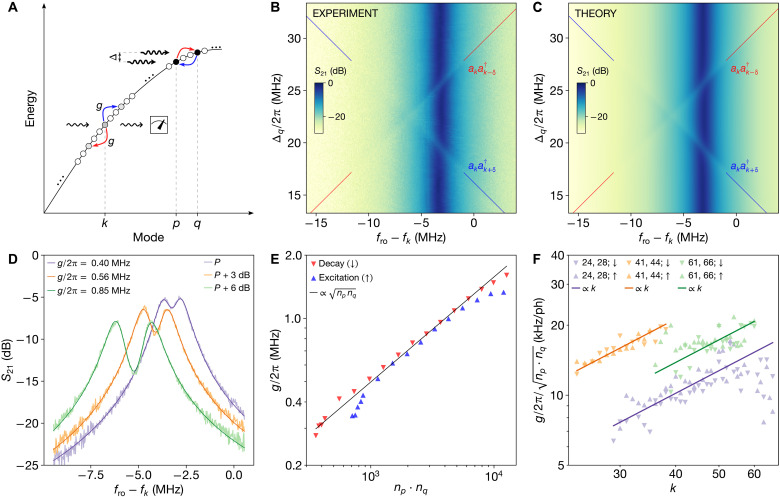
Matrix element scaling. (**A**) The mode of interest k couples to modes k±δ with the coupling strength g. By sweeping the detuning between two pumps, Δ/2π, resonant coupling can be achieved. (**B** and **C**) Experimental and theoretical transmission around mode k=29, when modes p=41 and q=44 are pumped. The colored guides indicate energy-conserving couplings between modes k with k+δ (blue) and k with k−δ (red). (**D**) Transmission for resonant coupling between k with k−δ for different pump powers. Darker lines are fits of the experimental data to eq. S25. Pump detuning dependence for these pump powers is shown in fig. S7. (**E**) Coupling strength g versus product of occupations of pumped modes, $n_{p}n_{q}$, closely follows a square root law. Different markers represent decay-like coupling (modes k and k−δ) and excitation-like coupling (modes k and k+δ). (**F**) Coupling strength g, normalized by the occupations of the pumped modes, increases with the readout mode k. In each dataset, pumped modes are fixed, and both decay-like and excitation-like couplings are measured (see legend).

In a weak-drive regime, the readout mode is resonantly coupled only to its two energy and momentum-conserving “nearest-neighbor” modes (Fig. 2A). The notion of nearest-neighbor modes emerges from the fact that a given mode k can now borrow or give away a momentum difference of δ=q−p, where p and q are numbers of the pumped modes. Thus, mode k can couple to modes k±δ. Energy conservation imposes the additional constraint of the frequency difference between pumps Δ/2π matching the energy difference between modes k and k±δ. Here, the curvature of the dispersion relation $\omega_{k}$ in Eq. 1 is crucial: $f_{k+\delta }-f_{k}\neq f_{k}-f_{k-\delta }$ (see Fig. 1C); thus, in general modes, k with k−δ and k with k+δ are coupled resonantly for different Δ. Experimentally, only one pump is detuned from the bare (undriven) mode frequency with the detuning $\Delta_{q}/2\pi >0$ and $\Delta_{p}=0$ or vice versa to prevent bifurcation (*47*–*49*).

In Fig. 2B, transmission is measured around mode k while sweeping pump detuning of the mode q. The measurement reveals additional features in the line shape of mode k, manifested as two straight lines in Fig. 2B. The occurrence of additional features in the line shape of mode k is consistent with effect of the interaction terms from Eq. 1 restricted to modes p, q, k and k±δ due to momentum and energy conservation

$$H_{\text{int}}^{\prime }=\hbar K\left[{\hat{a}}_{p}^{†}{\hat{a}}_{q}{\hat{a}}_{k+\delta }^{†}{\hat{a}}_{k}+{\hat{a}}_{q}^{†}{\hat{a}}_{p}{\hat{a}}_{k-\delta }^{†}{\hat{a}}_{k}+h.c.\right]$$ (2)

In the presence of strong drives, standard input-output theory and linearization techniques allow replacing operators of driven modes q and p by their expectation values, leading to effective beam splitter–like interaction $g{\hat{a}}_{k\pm \delta }^{†}{\hat{a}}_{k}$ (see the Supplementary Materials).

At a qualitative level, the beam-splitter interaction enables hybridization of modes k and k±δ that modifies the line shape. The conservation of energy in the process of scattering of photon from mode k+δ to the vicinity of mode k mediated via pumped modes gives the condition $f_{k+\delta }^{\prime }-f_{\text{ro}}=\Delta /2\pi$, where $f_{k+\delta }^{\prime }$ is the frequency of mode k+δ for a particular drive (here and below with prime, we emphasize the Kerr shift of mode frequency due to pumps). For the range of detunings of pump q where $f_{k+\delta }^{\prime }$ remains nearly constant, this condition parametrizes a line with slope −1, along which the feature is located. Analogously, scattering from mode k−δ gives a feature located along the line with slope +1 (see Fig. 2B). At the intersections of these lines with the resonance of mode k, two avoided crossings occur. At the quantitative level, an analytical prediction for transmission $S_{21}$ based on $H_{\text{int}}^{\prime }$ is shown in Fig. 2C. The theoretical description uses just two cuts at a fixed value of $\Delta_{q}$ from the experimental data to fit matrix elements, $f_{k}^{\prime }$, and linewidths and shows remarkable agreement with the experiment (for details, see the Supplementary Materials).

We now turn to the examination of the experimentally extracted mode coupling strength g of the effective interaction $g{\hat{a}}_{k\pm \delta }^{†}{\hat{a}}_{k}$. For the interaction in Eq. 2, in the limit of classical pumps of modes p and q with occupations $n_{p}$ and $n_{q}$, the coupling rate is proportional to the matrix element K and the geometric mean of occupations: $g=|K|\sqrt{n_{p}n_{q}}$ (see the Supplementary Materials). In Fig. 2D, a few measured transmission spectra of the avoided crossing are plotted together with their fits for different pump powers. The coupling g increases as a function of P, but slower than g∝P because, as pump power increases, modes p and q experience Kerr shifts, thus receiving fewer photons. To account for this effect, for each pump configuration, modes p and q were measured in reflection to extract the mode frequencies, as well as the internal, $\kappa_{i}$, and external, $\kappa_{\text{ex}}$, couplings. This allows the calculation of occupations $n_{p}$ and $n_{q}$ (*50*), and the results of this analysis are plotted in Fig. 2E for both avoided crossings, where g was extracted by fitting $S_{21}$ as in Fig. 2D. The data follow the $g\propto \sqrt{n_{p}n_{q}}$ trend, confirming that Eq. 1 is the correct starting point, and allowing the matrix element K/2π=16 kHz/ph to be extracted, which is in reasonable agreement with the expected value of 5 kHz/ph based on our independently determined device parameters and cryogenic insertion loss.

Next, the dependence of g on the readout mode k was investigated (Fig. 2F). The experimental $S_{21}$ data for the reported combinations of p,q,k were fit using the same procedure as in Fig. 2D. For each configuration, the pump power was chosen to remain in the regime g≲κ, where κ is the total linewidth, which offers a good trade-off between fit quality and the increasing deviation from the observed scaling $g\propto \sqrt{n_{p}n_{q}}$ at higher powers. For fixed modes being pumped, g increases as the readout mode number k is increased, and the corresponding power law is close to g∝k, giving insights into the microscopic mechanism responsible for the interaction term in Eq. 1 as follows. Josephson potential gives nonlinearity via higher order (gradient) expansion terms, with the matrix element that increases as $g\propto \sqrt{k\cdot \left(k\pm \delta \right)}\propto k$ (see the Supplementary Materials). The competing contribution to scattering from quantum phase slips—excitations that correspond to sudden winding of the phase by 2π—is expected to decrease with mode frequency and k (*43*, *51*, *52*). The experimentally observed dependence indicates that the gradient nonlinearity, as opposed to phase slips, is dominant at weak drives.

### Non-equilibrium photon cascades

**Fig. 3.**
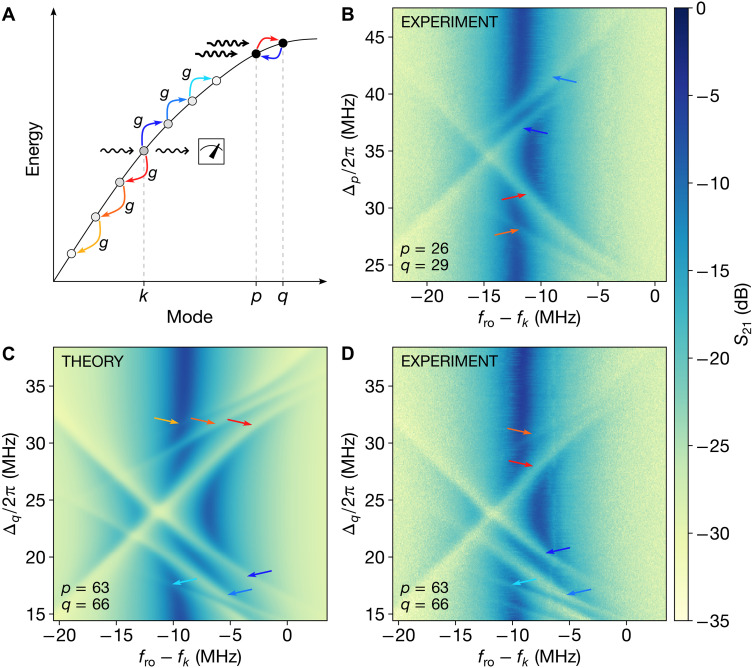
Cascades. (**A**) For sufficiently strong drives, mode k couples to modes separated by integer multiples of δ, i⋅δ, where i takes both positive and negative integer values. (**B** and **D**) Transmission measured around mode k=46 with two strong drives applied. Additional features appear for both pumping configurations: p,q<k (B) and p,q>k (D). (**C**) Numerically calculated transmission for the same configuration as in (D). In (B) to (D), colored arrows indicate processes schematically depicted in (A), where blue arrows represent excitation-like processes and red arrows represent decay-like processes.

After establishing multimode spectroscopy in the weak-driving regime and revealing the microscopic mechanism of nonlinearity in the effective Hamiltonian (*1*), we consider the more complex case of stronger drives illustrated in Fig. 3A. Stronger pumping of two modes p and q=p+δ enables coupling of an observed mode k to multiple further “neighbors” in a cascaded fashion: Mode k couples to k±δ, which in turn couples to k±2δ, and so on. To describe this cascaded coupling, we use the following notation: the coupling of mode k with mode k+iδ is referred to as the *i*th order process, where *i* is a nonzero integer. Using a similar energy conservation argument as above, we deduce that *i*th order processes require contribution from *i* photon scattering between pumped modes, resulting in a condition $f_{k+i\delta }^{\prime }-f_{\text{ro}}=i\cdot \Delta /2\pi$. For our pumping configuration where $f_{k+i\delta }^{\prime }$ stays nearly constant, this condition parametrizes the line with a slope of −1/i for p,q>k in ($f_{\text{ro}}-f_{k},\Delta_{q}/2\pi$) coordinates or 1/i for p,q<k in ($f_{\text{ro}}-f_{k},\Delta_{q}/2\pi$) coordinates. In Fig. 3 (B and D), in addition to lines with ±1 slope, extra features are observed for both p,q<k (Fig. 3B) and p,q>k (Fig. 3D). Qualitatively, these features can be labeled based on the knowledge of the mode frequencies in this driven configuration (colored arrows superimposed on Fig. 3, B and D, are in one-to-one correspondence with arrows in Fig. 3A).

By restricting and linearizing the full interaction Hamiltonian (*1*) to include only the nearest-neighbor mode couplings, e.g., k with k±δ, k±δ with k±2δ, and so on, we derive the analytical expression for the transmission spectrum (see discussion below in Eq. 2 and the Supplementary Materials). Figure 3C shows the numerically calculated transmission spectrum, closely matching the experimental results in Fig. 3D and revealing non-Lorentzian line shapes with multiple peaks. The agreement between theory and experiment provides strong evidence that cascaded photon decays are the dominant mechanism underlying the non-equilibrium response.

**Fig. 4.**
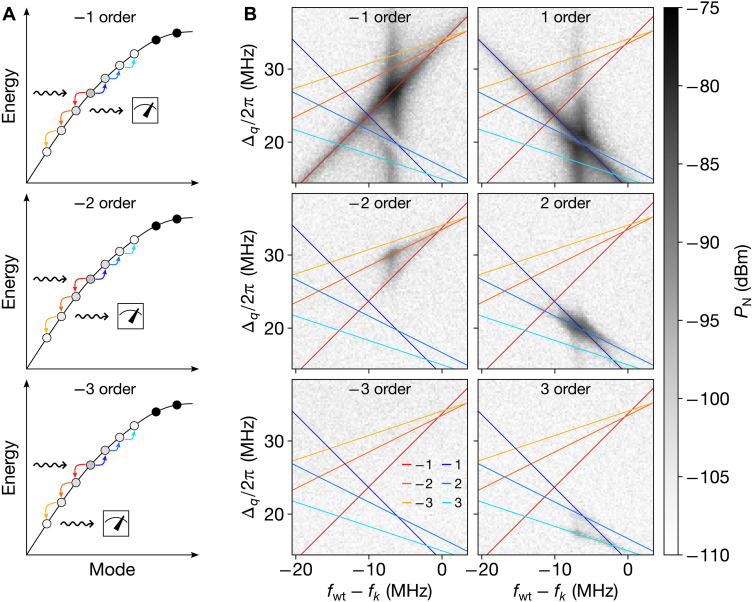
Direct observation of scattered photons. (**A**) Schematic of the noise measurement $P_{N}$ for cascaded scattering with orders −1, −2, and −3. Positive orders are measured analogously. (**B**) Noise power $P_{N}$ is measured for the processes with ∣i∣≤3. The intensity of the color represents the number of photons emitted from the cavity, on top of a background consisting of amplified input-referred added noise (of ≈ −103 dBm). The data in each panel are measured in the same pumping configuration, with the weak tone around k=46 and two pump tones p=63 and q=66. Colored guides [same colors as in (A)] show when the photon from the weak tone can be resonantly scattered to the mode k+i⋅δ.

This interpretation is further confirmed by directly detecting cascades in both lower and higher energy levels, as illustrated in Fig. 4A. Here, the noise power $P_{N}$ emitted from the device serves as a measure of mode occupation. Since the process of interest conserves energy and three tones are applied to the device input, the energy of the down- or up-scattered photon, ${\mathrm{hf}}_{N}$, corresponding to order *i*, is unambiguously determined. Thus, $P_{N}$ is measured in a narrow window of 270 Hz around the center frequency $f_{N}=f_{\text{wt}}+i\cdot \Delta /2\pi$, which is defined based on the pump tones and the frequency of the weak tone, $f_{\text{wt}}$. To ensure that the signal is easily resolvable, the power of the weak tone is slightly increased compared to the $S_{21}$ data presented earlier, resulting in a maximum mode k occupation of ~1 to 2 photons. It was verified that, at this power level of the weak tone, $S_{21}$ remains within the linear response regime.

In Fig. 4B, the noise power $P_{N}$ is plotted for cascaded scattering from mode k=46 when driving modes p=26 and q=29. Guidelines overlaid on the data indicate the regions where energy conservation is satisfied for scattering processes of a given order i. To determine the offsets of these lines, the mode frequencies for this driven configuration were independently identified. Features corresponding to ±1 order processes are highly pronounced in the respective subplots. For higher-order processes of ±2 and ±3, distinct features emerge when the weak tone frequency $f_{\text{wt}}$ coincides with the mode k (see fig. S9 for $S_{21}$ data measured in the same pumping configuration). This behavior is expected, as the occupation of mode k is highest when $f_{\text{wt}}$ is on resonance with mode k, and the number of scattered photons is proportional to $n_{k}$. For i=±1 orders, a decrease in $P_{N}$ is observed along the vertical feature when cascaded scattering with ∣i∣>1 becomes resonant with it, suggesting that higher-order resonant scattering can dominate over nonresonant processes toward “nearest-neighbors.” In such resonant regime, the measured noise power suggests that nearly all incoming photons are efficiently scattered, indicating near-complete, cascaded down-conversion. This is further supported by our estimates of conversion efficiency (see the Supplementary Materials). Analogous measurement with k>p,q is shown in fig. S8; theoretical calculation of the same quantity is presented in fig. S11.

### Photonic kinetics

We have demonstrated that even two mode pumping is capable of enhancing the interaction part of the Hamiltonian (*1*), leading to the non-Lorentzian line shapes from the cascaded redistribution of photons. Now, we turn to driving many modes, and studying the resulting near-continuum broadening of line shapes. This broadening emerges from intrinsic interaction-induced multimode scattering and is analogous to the quasiparticle lifetime of plasmons in one-dimensional quantum liquids (*32*, *43*) at equilibrium. Critically, in our experiment, the interplay of strong pumping, coupling of modes to waveguides, and nontrivial intrinsic relaxation due to multimode interactions drive the system far from equilibrium into a non-equilibrium steady state (NESS). In the simplest approximation, such NESS can be described by the occupation numbers of modes from Eq. 1, $n_{k}=\langle {\hat{a}}_{k}^{†}{\hat{a}}_{k}\rangle$, that is expected to be far from Bose-distribution function at the base temperature. Below, we will establish experimental ways of probing such steady state via broadening of line shapes, mode occupation numbers, and its redistribution in response to additional pumping.

**Fig. 5.**
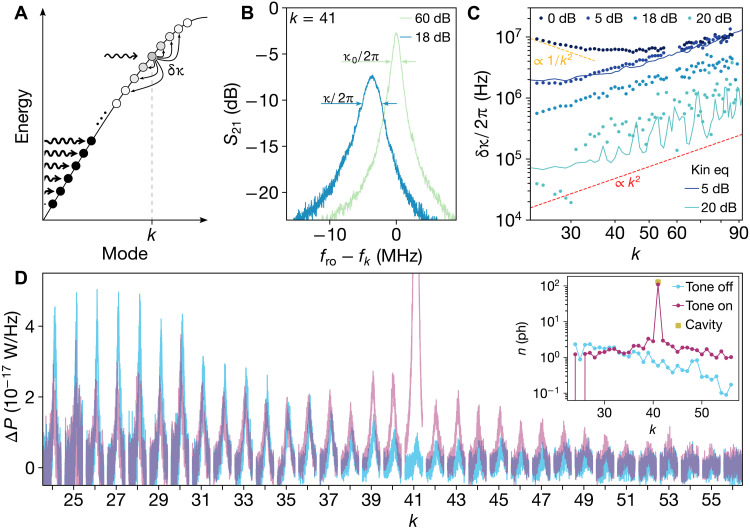
Incoherent broadband drive. (**A**) Schematic of the experiment. The first 13 modes are pumped using an incoherent broadband source, with power controlled by a variable attenuator. Modes within the measurement band are probed with a weak tone, and the excess decay δκ is extracted. (**B**) Under incoherent drive, the modes experience Kerr shift and an increase in linewidth. (**C**) Measured excess decay δκ as a function of noise power (points). Solid lines represent results from simulations of kinetic equation, and the dashed lines serve as a guide to the power law scaling of the decay. (**D**) Measurements of non-equilibrium occupations at 5-dB attenuation drive. The excess noise PSD, ΔP, is measured with and without a weak tone applied to mode 41. Inset shows the NESS distribution obtained by integrating the noise PSD from (D). Yellow marker indicates occupation of mode 41 calculated using cavity coupling parameters and the weak tone power.

To induce NESS and probe the continuous broadening of modes, we applied variable power broadband drive to a subset of modes k=1,…,13 (Fig. 5A). In this setup, a series of three amplifiers, acting as an incoherent drive source, is connected to the JJ-chain through a variable attenuator that controls the power of radiation entering the cryostat (see fig. S1). The maximum nominal attenuation (minimal power) of 60 dB gives the noise temperature of the radiation of ~300 K before entering the cryostat, with no observable change in mode frequencies or linewidths compared to the undriven case. For attenuations smaller than 30 dB, the modes exhibit a resolvable Kerr shift and at lower attenuations (higher noise power), and an increase in linewidth is detected (Fig. 5B). The linewidth change is quantified via excess decay $\delta \kappa =\kappa -\kappa_{0}$, where $\kappa_{0}$ represents the linewidth measured at 60-dB attenuation.

In Fig. 5C, the excess decay, δκ, is plotted for different readout modes k and several attenuations. For weak drive powers (high attenuations), the broadening increases with wave number k, consistent with the interaction terms in Eq. 1 as coming from gradient nonlinearities (*43*). The dependence on wave number is relatively weak, $\delta \kappa \propto k^{2}$, which contrasts with the theoretically predicted fourth-power scaling in the continuum regime (*43*). To understand this excess decay quantitatively, we use kinetic equations (*43*) to obtain the nonthermal distribution function in the NESS, $n_{k}$, and calculate the excess decay from the linearization of collision integral around the $n_{k}$. First, in Fig. 5C, we match the observed scalings of excess decay for attenuation of 20 dB, using a single fitting parameter that adjusts the occupation of the pumped modes (see the Supplementary Materials). At such low noise powers, the intrinsic scattering is relatively weak, so excess decay is not a smooth function of the mode number, both in theory and experiment. For intermediate-strength drives (e.g., 5 dB data), achieving a similar match between experiment and theory requires the introduction of additional internal loss for the pumped modes of ${\delta \kappa }_{i}=2\pi \times 6$ MHz. In the intermediate driving regime, the excess decay can exceed the bare linewidth, and excess loss becomes a smooth function of the mode number, $\delta \kappa \propto k^{2}$, signaling that intrinsic scattering from interactions is strongly enhanced. Last, at the strongest drives in Fig. 5C, we observe a nonmonotonic dependence of the excess decay δκ on mode number. Such qualitative change of excess decay cannot be explained by changes in NESS and signals presence of additional losses that are stronger for modes with low numbers, as anticipated with the introduction of ${\delta \kappa }_{i}$ at intermediate driving strength. We speculate that the excess decay may originate from interaction terms beyond the gradient nonlinearities included in Eq. 1, such as phase slips. Quantitatively, support for this hypothesis comes from the low-frequency power law, $\delta \kappa \propto k^{-2}$ shown in Fig. 5C, which is consistent with the expected scaling due to phase slips (*43*). An appealing possible picture is that the effective chain parameters are renormalized out of equilibrium, leading to a larger effective phase slip rate compared to the near-equilibrium case.

In addition to the excess decay, we also characterize the strongly driven regime by the occupation of modes in the NESS using noise measurements. We use the power spectral density (PSD) P measured within a 15-MHz band around modes with numbers k=24,…,56, both with the drive applied (5-dB attenuation) and without the drive (60 dB) (*53*, *54*). The difference, ΔP=P(5 dB)−P(60 dB), is plotted in Fig. 5D (“tone off”). Crucially, these data can be used to estimate the excess occupation on top of thermal distribution in the NESS. Ignoring thermal occupation at large mode numbers, we obtain the estimate of occupation numbers $n_{k}\approx \kappa_{\text{ex}}^{-1}\int \mathrm{df}\;\Delta P/\left(\mathrm{Ghf}\right)$, where the integration is performed over a window of 15 MHz around $f_{k}^{\prime }$, and G is the input-referred calibrated gain of the amplifier (fig. S10). This estimation has a systematic error of approximately a few decibels, and a more sophisticated method should be used to account for the losses of cryogenic components before the amplifier (*55*, *56*).

The occupation of mode numbers shown in Fig. 5D reveals at least one order of magnitude increase in $n_{k}$ compared to thermal distribution, even for modes with numbers k=30 that are far from pumped modes. Experimental imperfections, such as leakage outside the passband of the low-pass filter, cannot explain this excess occupation. However, the large enhancement of $n_{k}$ for k far away from pumped modes is consistent with intrinsic redistribution of photons due to interaction-induced scattering. Intuitively, photons injected by driving have enough opportunities to participate in multiple two photon-scattering events encoded by the interaction term in Eq. 1, which redistribute excitations from modes m,n→k,l. This causes an increase in the occupation of modes with k≫13 observed experimentally in Fig. 5D. The redistribution of excitations due to enhanced intrinsic scattering is also revealed by the response of NESS to additional tone applied to mode k=41. The “tone on” data in Fig. 5D shows the global change in PSD as a response to additional driving of mode 41. Converting these data to occupation numbers in the NESS, we observe that $n_{k}$ changes for all modes within the spectral range, witnessing nonlocal redistribution of excitations due to multiple interaction-induced scattering. In addition, additional driving of this mode verified the occupation estimates: The independently calculated occupation of mode 41 from the calibrated insertion loss (yellow marker in the inset of Fig. 5D) agrees well with the PSD measurement.

## DISCUSSION

In summary, we have established the quantized plasma modes in the Josephson junction chain as a practical realization of a driven, multimode nonlinear system. Using multimode microwave spectroscopy techniques, we characterized the Hamiltonian of the system in the regime of moderate interactions, directly observing cascaded couplings of plasma modes and experimentally identifying the microscopic origin of interaction term. Subjecting the system to strong drives, we created NESS, where drastic enhancement of intrinsic interactions becomes a limiting factor of plasma mode lifetimes. Direct visualization of nonthermal mode occupations and the resulting smooth mode broadenings reveals the realization of non-equilibrium strongly interacting liquid in a multimode superconducting resonator.

Although our system is quantum, it is important that many of our observables can be understood classically and do not rely on entanglement or negativity. The few-mode driving regime is described by linearized equations of motion, which can be viewed as classical equations for wave amplitudes. The steady state for many-mode drives is described by a kinetic equation for plasmon occupation number, which is similar to classical kinetics for wave action (*3*), albeit with some modifications due to quantum effects. We view the current work as a necessary starting point for future investigations of “more quantum” observables and regimes, such as intermode coherences, regime of small occupation numbers, and transient dynamics. The transient dynamics in particular can be strongly nonclassical even for weak nonlinearities (*57*).

Looking further ahead, our work opens a number of directions. We demonstrated that driving causes the broadening from momentum-conserving interactions δκ to exceed all other sources, suggesting realization of the hydrodynamic regime for plasmons. This invites detailed characterization of NESS and a search for emergence of the universal scaling regime as expected from the classical wave turbulence (*3*). Moreover, future work can explore the vast space of coherent and incoherent driving configurations, characterizing crossover between regimes of weak and strong multimode scattering, as well as potential transitions between different universal regimes.

In a different direction, coherent targeted driving of small subset of modes may allow to implement squeezing and creation of other nonclassical states and their characterization. In addition, the realization of nonreciprocal interactions between modes may lead to non-Hermitian lattice models (*58*). Last, mixing coherent and incoherent driving may enable studies of engineered wave turbulence with nonreciprocal interactions (*59*) and improve our understanding of microscopic dissipation mechanism in superconducting resonators.

More broadly, our work expands the experimental flexibility of Josephson arrays known for realizing different models (*60*–*62*) by adding powerful capabilities to induce crossover from a weakly interacting multimode system to a strongly coupled one-dimensional liquid. Combined with the flexible experimental toolbox of microwave control with circuit quantum electrodynamics (*63*), this expansion sets the stage for an exciting chapter of experimental exploration of strongly interacting quantum liquids.

## MATERIALS AND METHODS

### Device fabrication

The device was fabricated on a highly resistive silicon substrate using standard nanofabrication techniques. Electron-beam lithography (Raith EBPG5150) was performed on a resist stack consisting of LOR 5B/W/CSAR 62 with thicknesses of 440/40/100 nm, respectively (*64*). The high-contrast CSAR 62 layer was used to define the pattern on the W layer, which was subsequently etched using reactive ion etching with inductively coupled plasma (RIE-ICP) in SF_6_. In the exposed regions, the LOR 5B was removed with O_2_ ICP, followed by a short dip in diluted MF319 (MF319:H_2_O = 1:2) to eliminate any residual LOR 5B. After this step, the undercut was created, and suspended W bridges are formed. Double-angle Al deposition (25/50 nm at ±23°) was carried out in an ultrahigh-vacuum electron-beam evaporator (Plassys MEB550S2). After the first Al deposition, static oxidation was performed at 5 mbar for 5 min. Lift-off was completed in successive hot baths of dimethyl sulfoxide, acetone, and isopropyl alcohol.

### Microwave spectroscopy

Microwave measurements were carried out in an Oxford Triton dilution refrigerator with a base temperature of ~12 mK. The microwave wiring of the refrigerator is shown in fig. S1. For three-tone spectroscopy (Figs. 1 to 3), signals from two signal generators and a vector network analyzer were combined using a power splitter and a directional coupler. For noise measurements (Fig. 4), the output signal was directed to a spectrum analyzer while the rest of the measurement chain remained identical to that used in Figs. 1 to 3. Broadband pumping was implemented using a cascade of room-temperature amplifiers, with the total power entering the cryostat controlled by a variable attenuator. The insertion loss of the cryostat was measured using throughline calibration and, together with the loss of room temperature components, was used to infer device input referred power.

### Theory

We model the Josephson chain plasmons, interacting via quartic order nonlinearities, through the Hamiltonian in Eq. 1

$$H=\sum_{k>0}\hbar \omega_{k}{\hat{a}}_{k}^{†}{\hat{a}}_{k}+\sum_{\text{klmn}}\hbar K_{\text{klmn}}{\hat{a}}_{k}^{†}{\hat{a}}_{l}^{†}{\hat{a}}_{m}{\hat{a}}_{n}$$ (3)

The matrix element describing 2→2 scattering is given by

$$K_{\text{klmn}}=-\frac{E_{g}\pi^{2}}{4N^{3}}\sqrt{\text{klmn}}\sum_{s_{1},s_{2},s_{3}=\pm }\delta_{k+s_{1}l+s_{2}m+s_{3}n,0}$$ (4)

where k,l,m,n are mode numbers, $E_{g}$ is the ground charging energy, and N is the number of junctions.

To reproduce the weak drive kinetics, we use quantum Langevin equations and the input-output formalism, treating the two pumped modes as classical oscillating fields. Setting $i_{\text{max}}$ to be the maximum order of included multimode interaction, the transmission amplitude for the readout mode k is

$$S_{21}\left[\omega \right]=iS_{\text{fit}}\kappa_{\text{ex},k}{\left[{\left(\omega I-A_{\text{fit}}\right)}^{-1}\right]}_{i_{\text{max}}+1,i_{\text{max}}+1}e^{{\mathrm{i\varphi}}_{\text{fit}}}$$ (5)

with

$$\left\{\begin{array}{c} A_{\text{fit}}\left[n,n\right]=\omega_{k+\left(n-1-i_{\text{max}}\right)\delta }\left(1+\alpha_{\text{fit}}\right)-\left(n-1-i_{\text{max}}\right)\Delta -i\frac{\kappa_{\text{fit}}}{2}\\ A_{\text{fit}}\left[m,m-1\right]=A\left[m-1,m\right]=K_{p,p+\delta ,k+\left(m-1-i_{\text{max}}\right)\delta ,k+\left(m-2-i_{\text{max}}\right)\delta }n_{\text{fit}}^{\text{pump}} \end{array}\right.$$ (6)

The matrix indices n and m run in the range $n\in \left[1,2i_{\text{max}}+1\right]$, $m\in \left[2,2i_{\text{max}}+1\right]$. Δ is the difference between the pump frequencies, δ the distance between the pumped modes, and the five fitting parameters are emphasized by the corresponding subscript.

Moving to the strong multimode drive regime, we calculate the excess linewidth due to four-wave mixing, excluding self-decays, as

$$\begin{array}{cc} {\delta \kappa }_{k}= & \frac{\pi^{5}E_{g}^{2}}{16N^{6}}\sum_{p,q_{1},q_{2}}{\mathrm{kpq}}_{1}q_{2}\;\delta \left(\omega_{k}+\omega_{p}-\omega_{q_{1}}-\omega_{q_{2}}\right)\\ & \times \left[n_{p}\left(1+n_{q_{1}}+n_{q_{2}}\right)-n_{q_{1}}n_{q_{2}}\right]\sum_{s_{1},s_{2},s_{3}=\pm }\delta_{k+s_{1}p+s_{2}q_{1}+s_{3}q_{2},0} \end{array}$$ (7)

The occupation numbers correspond to the NESS of the system. This is obtained numerically by solving the kinetic equation

$${\dot{n}}_{k}=-\left(\kappa_{k}+{\delta \kappa }_{i,k}\right)\left(n_{k}-n_{k}^{\text{th}}\right)+{\alpha \kappa }_{\text{ex},k}n_{k}^{\text{flux}}+I_{\text{tot}}\left[k\right]$$ (8)

with $n^{\text{th}}$ the Bose-Einstein occupation, $n^{\text{flux}}$ the injected photons per unit frequency and time, κ the bare linewidth, and $\kappa_{\text{ex}}$ the coupling rate to each terminal. $I_{\text{tot}}\left[k\right]$ is the total collision integral

$$I_{\text{tot}}\left[k\right]=\sum_{p,q_{1},q_{2}}W_{p,k\to q_{1}q_{2}}\left\{\left(1+n_{p}\right)n_{q_{1}}n_{q_{2}}-n_{k}\left[n_{p}\left(1+n_{q_{1}}+n_{q_{2}}\right)-n_{q_{1}}n_{q_{2}}\right]\right\}$$ (9)

where the transition probability W is based on the above matrix element K. The conservation of energy during collisions is modeled as a Lorentzian with linewidth given by the sum of the linewidths of the four participating modes

$$\delta_{\gamma }\left(\omega \right)=\frac{1}{\pi }\frac{\gamma }{\gamma^{2}+\omega^{2}},\gamma =\sum_{j\in \left[k,p,q_{1},q_{2}\right]}\left(\kappa_{j}+{\delta \kappa }_{j}\right)/2,\omega =\omega_{q_{1}}+\omega_{q_{2}}-\omega_{k}-\omega_{p}$$ (10)

Such linewidths are updated every 10 time-steps of the evolution to include the excess linewidth ${\delta \kappa }_{j}$ due to the intrinsic nonlinear scattering. The parameter α=5 is a frequency-independent correction to the insertion loss of the fridge, determined by comparing the 20 dB data and simulations. For stronger drives (5 dB), we introduce an additional internal loss ${\delta \kappa }_{i,k}=6$ MHz for the driven modes (k=1,…,19).

## Supplementary Materials

### This PDF file includes:

Supplementary Text Figs. S1 to S11 Table S1 References
