# Supplementary material for: Non-equilibrium plasmon liquid in a Josephson junction chain
Source: Sci Adv. 2026 Feb 13;12(7):eady7222. doi: 10.1126/sciadv.ady7222 (PMC13267285; doi:10.1126/sciadv.ady7222)
Supplement: Supplementary file 1 — Supplementary Text Figs. S1 to S11 Table S1 References [file sciadv.ady7222_sm.pdf]

Science Advances
AAAS

Supplementary Materials for

Non-equilibrium plasmon liquid in a Josephson junction chain

Anton V. Bubis *et al.*

Corresponding author: Andrew P. Higginbotham, ahigginbotham@uchicago.edu

Sci. Adv. **12**, eady7222 (2026)
DOI: 10.1126/sciadv.ady7222

This PDF file includes:

Supplementary Text
Figs. S1 to S11
Table S1
References

JJ-chain parameters

First 173 modes were identified using the two-tone spectroscopy technique (see, for example, the Supplementary Information of Ref. (*27*)). To obtain frequencies unaffected by the Kerr shift due to pump, the pump power must be sufficiently low. Thus, both the pump power (1 dB step) and pump frequency (1 MHz step) were swept while recording S_{21} at a fixed f_{ro} (on resonance with the undriven mode 30). For each mode observed in the two-tone data, the lowest pump power was selected where a dip in the phase of S_{21} was detected above the noise floor. Mode frequencies obtained by this approach are plotted in (Fig. S2). The dispersion of the chain in the limit of $k \ll N$, where N is the number of junctions (for our chain $N = 13157$), is well-known (*26*):

$$2\pi f_k = \frac{v\mathbf{k}}{\sqrt{1+\left(\frac{v\mathbf{k}}{\omega_{\mathrm{p}}}\right)^2}}, \quad \mathbf{k} = k\frac{\pi}{L}. \tag{S1}$$

By fitting to Eq. (S1), the speed of light v and the plasma frequency of a single junction ω_{p} were extracted.

A complete description of the JJ-chain requires three independent energy scales: the Josephson energy E_{J}, the junction charging energy E_{C}, and the ground charging energy E_{g} (*26*). However, v and ω_{p} depend on the products $E_{\mathrm{J}}E_{\mathrm{g}}$ and $E_{\mathrm{J}}E_{\mathrm{C}}$, meaning that the chain parameters cannot be independently extracted. Additional measurements are necessary to determine relevant energies independently, for example, through DC measurements of the chain (see Supplementary Information in Ref. (*28*)).

To fix device parameters we calculate E_g based on the device geometry, and use the fit to determine the remaining unknown parameters E_J and E_C. E_g is found from standard transmission line formulas (*65, 66*). To check the result, we also simulated our device geometry in Sonnet, finding a value that agrees with the analytical formula to within 1%. The standard error of the fit plasmon speed v and the plasma frequency ω_{p} are also less than 1%, making it tempting to infer an overall parameter error of only a few percent. However, caution in interpreting the standard errors is needed because the measured dispersion (Fig. S2B) shows a systematic discrepancy with the theory.

As a rough consistency check, we also estimate the capacitance of a single junction from its geometry together with a nominal specific capacitance of $Al/AlO_x/Al$ junctions, using the typical range 50-100 fF/µm^2 (*67, 68*), determining the remaining two parameters E_g and E_J from the fit. It is comforting that the rough consistency check overlaps with the more precisely-determined value based on device geometry. Both approaches follow the methodology outlined in Ref. (*27*).

Derivation of the effective Hamiltonian and matrix element

In this Section, we derive the matrix element K responsible for the beam-splitter-like interaction. Our starting point is the following Luttinger liquid quadratic Hamiltonian, describing the JJ chain in the continuum (*43*)

and in the absence of nonlinearities,

$$H_0 = \frac{1}{2\pi^2}\int_0^N dx\left[\frac{v_s}{K_g}|\partial_x\phi(x)|^2 + v_s K_g \pi^2 |\partial_x\theta(x)|^2\right]. \tag{S2}$$

Here $K_{\mathrm{g}} = \sqrt{E_{\mathrm{J}}/(2E_{\mathrm{g}})}$, $v_s = \sqrt{2E_{\mathrm{J}}E_{\mathrm{g}}}$, and N is the number of Josephson junctions. $\theta(x)$ is the superconducting phase field, and $\phi(x)$, related to the number of Cooper pairs, is its canonically conjugated. We set $\hbar = 1$, and express distances in units of the junctions' spacing L/N, where L is the chain's size. With this choice, $x = r\frac{N}{L}$, where r has dimension of length. The fields ϕ and θ can be expanded on bosonic operators describing the plasmonic modes sustained by the chain,

$$\theta(x) = \mathrm{i}\left(\frac{2E_{\mathrm{g}}}{E_{\mathrm{J}}}\right)^{1/4}\frac{1}{\sqrt{N}}\sum_{\mathbf{k}>0}\frac{1}{\sqrt{\mathbf{k}}}\sin(\mathbf{k}x)(\hat{a}^\dagger_{\mathbf{k}} - \hat{a}_{\mathbf{k}}), \tag{S3}$$

$$\phi(x) = \pi\left(\frac{E_{\mathrm{J}}}{2E_{\mathrm{g}}}\right)^{1/4}\frac{1}{\sqrt{N}}\sum_{\mathbf{k}>0}\frac{1}{\sqrt{\mathbf{k}}}\cos(\mathbf{k}x)(\hat{a}^\dagger_{\mathbf{k}} + \hat{a}_{\mathbf{k}}). \tag{S4}$$

$\mathbf{k}$ is the quasimomentum, which can be quantized as $\mathbf{k} = \pi k/N$, where $k = 1, 2, \ldots$ are the corresponding mode numbers. The above expansions rely on the plasmonic wavefunctions under open boundary conditions, which take the following form

$$\psi_k(x) = \sqrt{\frac{2}{N}}\sin\left(\frac{\pi k x}{N}\right). \tag{S5}$$

After substitution of Eqs. (S3,S4) in Eq. (S2), we get the second quantized Hamiltonian

$$H_0 = \sum_{k>0} v_s k\left(\hat{a}^\dagger_k\hat{a}_k + \frac{1}{2}\right) = \sum_{k>0}\underbrace{\sqrt{2E_{\mathrm{g}}E_{\mathrm{J}}}\frac{\pi}{N}k}_{\omega_k}\left(\hat{a}^\dagger_k\hat{a}_k + \frac{1}{2}\right), \tag{S6}$$

which allows us to identify mode frequencies in our notations, $\omega_k \approx v_s k$. Notice that Eq. (S2) was already assuming a linearized plasmonic dispersion. If we also apply second quantization to the leading *nonlinear* term from the expansion of the cosine Josephson potential,

$$H_{\mathrm{int}} = -\frac{E_{\mathrm{J}}}{24}\int_0^N dx\,(\partial_x\theta)^4, \tag{S7}$$

we get

$$H_{\mathrm{int}} = -\frac{E_{\mathrm{g}}\pi^2}{96N^3}\sum_{k,l,m,n>0}\sqrt{klmn}(\hat{a}^\dagger_k - \hat{a}_k)(\hat{a}^\dagger_l - \hat{a}_l)(\hat{a}^\dagger_m - \hat{a}_m)(\hat{a}^\dagger_n - \hat{a}_n)\sum_{s_1,s_2,s_3=\pm}\delta_{k+s_1 l+s_2 m+s_3 n,0}. \tag{S8}$$

We have verified that $1 \to 3$ scattering – arising from $\hat{a}_k\hat{a}_l\hat{a}_m\hat{a}_n^\dagger$ – is energetically much less favorable than $2 \to 2$, though arising at the same nonlinear order. For this reason we neglect it in our analysis. The four-wave mixing matrix element is

$$K_{klmn} = \langle 0|\hat{a}_k\hat{a}_l\hat{H}_{\rm int}\hat{a}_m^\dagger\hat{a}_n^\dagger|0\rangle = -\frac{E_{\rm g}\pi^2}{4N^3}\sqrt{klmn}\sum_{s_1,s_2,s_3=\pm}\delta_{k+s_1l+s_2m+s_3n,0}. \tag{S9}$$

Notice that, for plasma modes in a confined system, quasimomentum is conserved only modulo sign, as each standing wave supports two components propagating in opposite directions. The expression for K_{klmn} determines the nonlinear term in the full Hamiltonian in Eq. (1) in the main text (obtained after restriction to boson-number conserving terms only), and its restriction to the momentum and energy conserving processes results in Eq. (2) and will be used below to calculate the analytical predictions for the transmission.

We have verified that the matrix element associated with sixth order nonlinearities is strongly suppressed compared to that of the fourth order. This justifies restricting our analysis to the fourth order interaction. Furthermore, when investigating the excess linewidths (Sec. SM V), we checked that the extra broadening due to sixth order processes is negligible.

Quantum Langevin equations

Let us start from the Hamiltonian describing JJ-chain expanded up to the fourth order (see Eq. (2)):

$$H = H_0 + H_{\rm int} = \sum_{k>0}\hbar\omega_k\hat{a}_k^\dagger\hat{a}_k + \sum_{klmn}\hbar K_{klmn}\hat{a}_k^\dagger\hat{a}_l^\dagger\hat{a}_m\hat{a}_n, \tag{S10}$$

where $H_{\rm int}$ is a nonlinear contribution originated from the expansion of the Josephson cosine potential, and it contains all permutations of four bosonic operators (quartic terms). To illustrate the four-wave mixing process of the Hamiltonian Eq. (S10), let us focus on the process $i, p \leftrightarrow j, q$:

$$H = \sum_{k=i,j,p,q}\hbar\omega_k\hat{a}_k^\dagger\hat{a}_k + \hbar K_{ijpq}\left(\hat{a}_i^\dagger\hat{a}_j\hat{a}_p^\dagger\hat{a}_q + \hat{a}_i\hat{a}_j^\dagger\hat{a}_p\hat{a}_q^\dagger\right),$$

where we leave just four modes of interest and assume that mode frequencies ω_k already include Kerr shifts (highlighted with prime index in the main text). For both pumps of modes p and q we can use classical fields $A_p e^{-i\omega_p^{\rm pump}t}$ and $A_q e^{-i\omega_q^{\rm pump}t}$, respectively. After this substitution, the Hamiltonian reads:

$$H = \hbar\left(\omega_i\hat{a}_i^\dagger\hat{a}_i + \omega_j\hat{a}_j^\dagger\hat{a}_j + g\hat{a}_i^\dagger\hat{a}_j e^{-i\Delta t} + g^*\hat{a}_i\hat{a}_j^\dagger e^{i\Delta t}\right),$$

where $g = K_{ijpq}A_p^* A_q$, $\Delta = \omega_q^{\text{pump}} - \omega_p^{\text{pump}}$. Now we need to solve quantum Langevin equations for modes $k = i, j$:

$$\frac{d\hat{a}_k}{dt} = -\frac{\mathrm{i}}{\hbar}\left[\hat{a}_k, H\right] - \frac{\kappa_{\mathrm{L},k} + \kappa_{\mathrm{R},k} + \kappa_{\mathrm{i},k}}{2}\hat{a}_k + \sqrt{\kappa_{\mathrm{L},k}}\hat{a}_{\mathrm{IN,L}} + \sqrt{\kappa_{\mathrm{R},k}}\hat{a}_{\mathrm{IN,R}} + \sqrt{\kappa_{\mathrm{i},k}}\hat{f}_{\mathrm{IN}}.$$

Here $\hat{a}_{\mathrm{IN,L}}, \hat{a}_{\mathrm{IN,R}}$ are input fields on the left and right side of the cavity, and $\hat{f}_{\mathrm{IN}}$ is an effective Langevin force which describes all loss mechanisms. Assuming for simplicity mode independent linewidth $\kappa_{(\mathrm{R,L}),k} = \kappa_{\mathrm{ex}}$, $\kappa_{\mathrm{i},k} = \kappa_{\mathrm{i}}$ and $\kappa = 2\kappa_{\mathrm{ex}} + \kappa_{\mathrm{i}}$ we obtain:

$$\frac{d\hat{a}_i}{dt} = -\mathrm{i}\omega_i\hat{a}_i - \mathrm{i}g\hat{a}_j e^{-\mathrm{i}\Delta t} - \frac{\kappa}{2}\hat{a}_i + \sqrt{\kappa_{\mathrm{ex}}}\hat{a}_{\mathrm{IN,L}} + \sqrt{\kappa_{\mathrm{ex}}}\hat{a}_{\mathrm{IN,R}} + \sqrt{\kappa_{\mathrm{i}}}\hat{f}_{\mathrm{IN}}, \tag{S11}$$

$$\frac{d\hat{a}_j}{dt} = -\mathrm{i}\omega_j\hat{a}_j - \mathrm{i}g^*\hat{a}_i e^{\mathrm{i}\Delta t} - \frac{\kappa}{2}\hat{a}_j + \sqrt{\kappa_{\mathrm{ex}}}\hat{a}_{\mathrm{IN,L}} + \sqrt{\kappa_{\mathrm{ex}}}\hat{a}_{\mathrm{IN,R}} + \sqrt{\kappa_{\mathrm{i}}}\hat{f}_{\mathrm{IN}}. \tag{S12}$$

After taking Fourier transform of Eqs. (S11)-(S12):

$$-\mathrm{i}\omega\hat{a}_i[\omega] = -\mathrm{i}\omega_i\hat{a}_i[\omega] - \mathrm{i}g\hat{a}_j[\omega - \Delta] - \frac{\kappa}{2}\hat{a}_i[\omega] + \sqrt{\kappa_{\mathrm{ex}}}\hat{a}_{\mathrm{IN,L}}[\omega] + \sqrt{\kappa_{\mathrm{ex}}}\hat{a}_{\mathrm{IN,R}}[\omega] + \sqrt{\kappa_{\mathrm{i}}}\hat{f}_{\mathrm{IN}}[\omega],$$

$$-\mathrm{i}\omega\hat{a}_j[\omega] = -\mathrm{i}\omega_j\hat{a}_j[\omega] - \mathrm{i}g^*\hat{a}_i[\omega + \Delta] - \frac{\kappa}{2}\hat{a}_j[\omega] + \sqrt{\kappa_{\mathrm{ex}}}\hat{a}_{\mathrm{IN,L}}[\omega] + \sqrt{\kappa_{\mathrm{ex}}}\hat{a}_{\mathrm{IN,R}}[\omega] + \sqrt{\kappa_{\mathrm{i}}}\hat{f}_{\mathrm{IN}}[\omega].$$

Finally, introducing notations:

$$\chi_i^{(3)}[\omega] = \frac{1}{\kappa/2 - \mathrm{i}(\omega - \omega_j - \Delta)},$$

$$\chi_j^{(3)}[\omega] = \frac{1}{\kappa/2 - \mathrm{i}(\omega - \omega_i + \Delta)},$$

$$\chi_k[\omega] = \frac{1}{\kappa/2 - \mathrm{i}(\omega - \omega_k) + |g|^2\chi_k^{(3)}[\omega]},$$

we obtain:

$$\hat{a}_i[\omega] = \chi_i[\omega]\left[\left(\sqrt{\kappa_{\mathrm{ex}}}\hat{a}_{\mathrm{IN,L}} + \sqrt{\kappa_{\mathrm{ex}}}\hat{a}_{\mathrm{IN,R}} + \sqrt{\kappa_{\mathrm{i}}}\hat{f}_{\mathrm{IN}}\right)[\omega] - \mathrm{i}g\chi_i^{(3)}[\omega]\left(\sqrt{\kappa_{\mathrm{ex}}}\hat{a}_{\mathrm{IN,L}} + \sqrt{\kappa_{\mathrm{ex}}}\hat{a}_{\mathrm{IN,R}} + \sqrt{\kappa_{\mathrm{i}}}\hat{f}_{\mathrm{IN}}\right)[\omega - \Delta]\right],$$

$$\hat{a}_j[\omega] = \chi_j[\omega]\left[\left(\sqrt{\kappa_{\mathrm{ex}}}\hat{a}_{\mathrm{IN,L}} + \sqrt{\kappa_{\mathrm{ex}}}\hat{a}_{\mathrm{IN,R}} + \sqrt{\kappa_{\mathrm{i}}}\hat{f}_{\mathrm{IN}}\right)[\omega] - \mathrm{i}g^*\chi_j^{(3)}[\omega]\left(\sqrt{\kappa_{\mathrm{ex}}}\hat{a}_{\mathrm{IN,L}} + \sqrt{\kappa_{\mathrm{ex}}}\hat{a}_{\mathrm{IN,R}} + \sqrt{\kappa_{\mathrm{i}}}\hat{f}_{\mathrm{IN}}\right)[\omega + \Delta]\right].$$

Using boundary conditions:

$$\hat{a}_{\mathrm{IN,R}} + \hat{a}_{\mathrm{OUT,R}} = \sqrt{\kappa_{\mathrm{ex}}}\hat{a}_i + \sqrt{\kappa_{\mathrm{ex}}}\hat{a}_j,$$

$$\hat{a}_{\mathrm{IN,L}} + \hat{a}_{\mathrm{OUT,L}} = \sqrt{\kappa_{\mathrm{ex}}}\hat{a}_i + \sqrt{\kappa_{\mathrm{ex}}}\hat{a}_j,$$

we can express averaged output field $\langle \hat{a}_{\mathrm{OUT,L}} \rangle$ via input $\langle \hat{a}_{\mathrm{IN,R}} \rangle$:

$$S_{21}[\omega] = \frac{\langle \hat{a}_{\mathrm{OUT,L}} \rangle[\omega]}{\langle \hat{a}_{\mathrm{IN,R}} \rangle[\omega]} = \kappa_{\mathrm{ex}}(\chi_i[\omega] + \chi_j[\omega]) \approx \frac{\kappa_{\mathrm{ex}}}{\kappa/2 - \mathrm{i}(\omega - \omega_i) + \frac{|g|^2}{\kappa/2 - \mathrm{i}(\omega - \omega_j - \Delta)}}, \tag{S13}$$

where in the last expression we ignored the small contribution from $\chi_j[\omega]$, because we are interested in a solution in the vicinity of mode i. This expression is used to extract matrix element from experimental data e.g. in Fig. 2D of the maintext.

Generalization of quantum Langevin equations to multiplets of modes

This Section is devoted to the theoretical description of the cascaded process shown in Fig. 3 of the main text. In this process, the excitation down or up-scatters from mode k to mode $k \mp i\delta$, and a simultaneous up-scattering or down-scattering from mode p to mode $p \pm \delta$ occurs i times in order to satisfy momentum conservation. Schematically this process may be written as:

$$k \to k - i\delta, \qquad [p \to p + \delta]^i \tag{S14}$$

$$k \to k + i\delta, \qquad [p + \delta \to p]^i. \tag{S15}$$

Here $p, q = p + \delta$ are the pumped modes, k is the readout mode, and $i \in [1, i_{\mathrm{max}}]$ is a positive integer. The upper bound i_{max} sets to which order the multi-mode interaction is included.

In order to account for processes in Eqs. (S14)-(S15), we can restrict the general Hamiltonian (Eq. (1) in the main text) to the following set of modes

$$\mathcal{M} = \{p, p + \delta, k - i_{\mathrm{max}}\delta, k - (i_{\mathrm{max}} - 1)\delta, \dots, k - \delta, k, k + \delta, \dots, k + (i_{\mathrm{max}} - 1)\delta, k + i_{\mathrm{max}}\delta\}. \tag{S16}$$

The Hamiltonian describing such set reads (we set $\hbar = 1$)

$$H_{\mathcal{M}} = \sum_{j \in \mathcal{M}} \omega_j \hat{a}_j^\dagger \hat{a}_j + \\ A_p e^{-\mathrm{i}\omega_p^{\mathrm{pump}} t} A_{p+\delta}^* e^{\mathrm{i}\omega_{p+\delta}^{\mathrm{pump}} t} \sum_{i=1}^{i_{\mathrm{max}}} \left[K_{p,p+\delta,k+(i-1)\delta,k+i\delta} \hat{a}_{k+(i-1)\delta}^\dagger \hat{a}_{k+i\delta} + K_{p,p+\delta,k-(i-1)\delta,k-i\delta} \hat{a}_{k-i\delta}^\dagger \hat{a}_{k-(i-1)\delta} \right] + \mathrm{h.c.}, \tag{S17}$$

where we have replaced the two pumped modes by classical fields. The Langevin equations for all the other

modes in $\mathcal{M}$ reads (we omit the Langevin force $\hat{f}_{\rm IN}$ for brevity)

$$\begin{aligned}
\dot{\hat{a}}_{k-i_{\max}\delta} &= -\mathrm{i}\omega_{k-i_{\max}\delta}\hat{a}_{k-i_{\max}\delta} - \mathrm{i}K_{p,p+\delta,k-i_{\max}\delta,k-(i_{\max}-1)\delta}A_pA^*_{p+\delta}e^{-\mathrm{i}(\omega_p^{\rm pump}-\omega_{p+\delta}^{\rm pump})t}\hat{a}_{k-(i_{\max}-1)\delta} - \frac{\kappa_{k-i_{\max}\delta}}{2}\hat{a}_{k-i_{\max}\delta},\\
\dot{\hat{a}}_{k-i\delta} &= -\mathrm{i}\omega_{k-i\delta}\hat{a}_{k-i\delta} - \mathrm{i}K_{p,p+\delta,k-i\delta,k-(i-1)\delta}A_pA^*_{p+\delta}e^{-\mathrm{i}(\omega_p^{\rm pump}-\omega_{p+\delta}^{\rm pump})t}\hat{a}_{k-(i-1)\delta}\\
&\quad -\mathrm{i}K_{p,p+\delta,k-(i+1)\delta,k-i\delta}A^*_pA_{p+\delta}e^{\mathrm{i}(\omega_p^{\rm pump}-\omega_{p+\delta}^{\rm pump})t}\hat{a}_{k-(i+1)\delta} - \frac{\kappa_{k-i\delta}}{2}\hat{a}_{k-i\delta}, \qquad i\neq i_{\max}\\
\dot{\hat{a}}_{k} &= -\mathrm{i}\omega_k\hat{a}_k - \mathrm{i}K_{p,p+\delta,k,k+\delta}A_pA^*_{p+\delta}e^{-\mathrm{i}(\omega_p^{\rm pump}-\omega_{p+\delta}^{\rm pump})t}\hat{a}_{k+\delta} - \mathrm{i}K_{p,p+\delta,k-\delta,k}A^*_pA_{p+\delta}e^{\mathrm{i}\omega_p^{\rm pump}t}e^{-\mathrm{i}\omega_{p+\delta}^{\rm pump}t}\hat{a}_{k-\delta} - \frac{\kappa_k}{2}\hat{a}_k,\\
\dot{\hat{a}}_{k+i\delta} &= -\mathrm{i}\omega_{k+i\delta}\hat{a}_{k+i\delta} - \mathrm{i}K_{p,p+\delta,k+i\delta,k+(i-1)\delta}A^*_pA_{p+\delta}e^{\mathrm{i}(\omega_p^{\rm pump}-\omega_{p+\delta}^{\rm pump})t}\hat{a}_{k+(i-1)\delta}\\
&\quad -\mathrm{i}K_{p,p+\delta,k+(i+1)\delta,k+i\delta}A_pA^*_{p+\delta}e^{-\mathrm{i}(\omega_p^{\rm pump}-\omega_{p+\delta}^{\rm pump})t}\hat{a}_{k+(i+1)\delta} - \frac{\kappa_{k+i\delta}}{2}\hat{a}_{k+i\delta}, \qquad i\neq i_{\max}\\
\dot{\hat{a}}_{k+i_{\max}\delta} &= -\mathrm{i}\omega_{k+i_{\max}\delta}\hat{a}_{k+i_{\max}\delta} - \mathrm{i}K_{p,p+\delta,k+i_{\max}\delta,k+(i_{\max}-1)\delta}A^*_pA_{p+\delta}e^{\mathrm{i}(\omega_p^{\rm pump}-\omega_{p+\delta}^{\rm pump})t}\hat{a}_{k+(i_{\max}-1)\delta} - \frac{\kappa_{k+i_{\max}\delta}}{2}\hat{a}_{k+i_{\max}\delta}.
\end{aligned}$$

We introduce the notation

$$\Delta = \omega_{p+\delta}^{\rm pump} - \omega_p^{\rm pump} \tag{S18}$$

for the energy difference between pumped modes, and move to the rotating frame

$$\hat{a}_{k\pm i\delta} = \hat{b}_{k\pm i\delta}e^{-i\Omega_{k\pm i\delta}t}, \qquad i\in[1,i_{\max}], \tag{S19}$$

where the Ω's have to be chosen such that the equations of motion become time-independent. The readout mode is left unchanged, $\hat{a}_k = \hat{b}_k$. We observe that the physically motivated choice of the frequency $\Omega_{k\pm i\delta}$ equal to the i times the energy difference between two pumped modes removes the time dependence,

$$\Omega_{k\pm i\delta} = \pm i\Delta, \qquad i\in[1,i_{\max}]. \tag{S20}$$

Using the above expression for $\Omega_{k\pm i\delta}$ and rotating frame (S19), and substituting $A_pA^*_{p+\delta} = A^*_pA_{p+\delta} = \sqrt{n_pn_{p+\delta}}$ (n_p and $n_{p+\delta}$ are the number of photons in the two pumped modes), we obtain equations without explicit time dependence,

$$\begin{aligned}
\dot{\hat{b}}_k &= -\mathrm{i}\omega_k\hat{b}_k - \mathrm{i}K_{p,p+\delta,k,k+\delta}\sqrt{n_pn_{p+\delta}}\,\hat{b}_{k+\delta} - \mathrm{i}K_{p,p+\delta,k-\delta,k}\sqrt{n_pn_{p+\delta}}\,\hat{b}_{k-\delta} - \frac{\kappa_k}{2}\hat{b}_k,\\
\dot{\hat{b}}_{k+i\delta} &= -\mathrm{i}(\omega_{k+i\delta} - i\Delta)\hat{b}_{k+i\delta} - \mathrm{i}K_{p,p+\delta,k+(i-1)\delta,k+i\delta}\sqrt{n_pn_{p+\delta}}\,\hat{b}_{k+(i-1)\delta}\\
&\quad - \mathrm{i}K_{p,p+\delta,k+(i+1)\delta,k+i\delta}\sqrt{n_pn_{p+\delta}}\,\hat{b}_{k+(i+1)\delta} - \frac{\kappa_{k+i\delta}}{2}\hat{b}_{k+i\delta}, \quad \text{for} \quad i\neq i_{\max}\\
\dot{\hat{b}}_{k+i_{\max}\delta} &= -\mathrm{i}(\omega_{k+i_{\max}\delta} - i_{\max}\Delta)\hat{b}_{k+i_{\max}\delta} - \mathrm{i}K_{p,p+\delta,k+i_{\max}\delta,k+(i_{\max}-1)\delta}\sqrt{n_pn_{p+\delta}}\,\hat{b}_{k+(i_{\max}-1)\delta} - \frac{\kappa_{k+i_{\max}\delta}}{2}\hat{b}_{k+i_{\max}\delta}.
\end{aligned}$$

We reported the last three equations, but the strategy is analogous for the first two. The resulting set of all equations can be compactly represented in a matrix form,

$$\frac{d}{dt}\begin{pmatrix} \hat{b}_{k-i_{\max}\delta} \\ \hat{b}_{k-(i_{\max}-1)\delta} \\ \vdots \\ \hat{b}_{k-\delta} \\ \hat{b}_k \\ \hat{b}_{k+\delta} \\ \vdots \\ \hat{b}_{k+(i_{\max}-1)\delta} \\ \hat{b}_{k+i_{\max}\delta} \end{pmatrix} = -\mathrm{i}\mathbb{A}\begin{pmatrix} \hat{b}_{k-i_{\max}\delta} \\ \hat{b}_{k-(i_{\max}-1)\delta} \\ \vdots \\ \hat{b}_{k-\delta} \\ \hat{b}_k \\ \hat{b}_{k+\delta} \\ \vdots \\ \hat{b}_{k+(i_{\max}-1)\delta} \\ \hat{b}_{k+i_{\max}\delta} \end{pmatrix}. \tag{S21}$$

The matrix $\mathbb{A}$ has tridiagonal form with entries

$$\begin{cases} \mathbb{A}[n,n] = \omega_{k+(n-1-i_{\max})\delta} - (n-1-i_{\max})\Delta - \mathrm{i}\frac{\kappa_{k+(n-1-i_{\max})\delta}}{2} \\ \mathbb{A}[m,m-1] = \mathbb{A}[m-1,m] = K_{p,p+\delta,k+(m-1-i_{\max})\delta,k+(m-2-i_{\max})\delta}\sqrt{n_p n_{p+\delta}} \end{cases}, \tag{S22}$$

where matrix indices n and m run in the range $n \in [1, 2i_{\max}+1]$, $m \in [2, 2i_{\max}+1]$. Moving to the Fourier space, where the time derivative becomes a factor $-i\omega$, and coupling the probed mode k to the incoming radiation via an additional term $\sqrt{\kappa_{\mathrm{ex},k}}(\hat{a}_{\mathrm{IN,R}} + \hat{a}_{\mathrm{IN,L}})$, where $\hat{a}_{\mathrm{IN,R/L}}$ is the input field on either side of the cavity, we express $\hat{b}_k$ via incoming fields as:

$$\hat{b}_k = \mathrm{i}\sqrt{\kappa_{\mathrm{ex},k}}(\hat{a}_{\mathrm{IN,R}} + \hat{a}_{\mathrm{IN,L}})[(\omega\mathbb{I} - \mathbb{A})^{-1}]_{i_{\max}+1,i_{\max}+1}. \tag{S23}$$

The subscripts select the relevant entry of the inverted matrix $(\omega\mathbb{I} - \mathbb{A})^{-1}$, and $\mathbb{I}$ is the identity matrix of size $(2i_{max}+1)$. Using boundary conditions similar to the previous Section, we finally obtain

$$S_{21}[\omega] = \mathrm{i}\kappa_{\mathrm{ex},k}\left[(\omega\mathbb{I} - \mathbb{A})^{-1}\right]_{i_{\max}+1,i_{\max}+1}. \tag{S24}$$

Application for coupling to both nearest-neighbors

Eq. (S24) can be used to obtain a generalization of Eq. (S13) which takes into account both processes $k \to k+\delta$ and $k \to k-\delta$ (i.e. $i_{\max} = 1$). This is useful to reproduce experimental data with low pump power, where just two avoided crossings are observed (see Fig. 2B). In Eq. (S24) we set $i_{\max} = 1$:

$$S_{21}[\omega] = \mathrm{i}\kappa_{\mathrm{ex},k}\left[(\omega\mathbb{I} - \mathbb{A})^{-1}\right]_{22},$$

with

$$\mathbb{A} = \begin{pmatrix} \omega_{k-\delta} + \Delta - \frac{\mathrm{i}\kappa_{k-\delta}}{2} & K_{p,p+\delta,k,k-\delta}\sqrt{n_p n_{p+\delta}} & 0 \\ K_{p,p+\delta,k,k-\delta}\sqrt{n_p n_{p+\delta}} & \omega_k - \frac{\mathrm{i}\kappa_k}{2} & K_{p,p+\delta,k,k+\delta}\sqrt{n_p n_{p+\delta}} \\ 0 & K_{p,p+\delta,k,k-\delta}\sqrt{n_p n_{p+\delta}} & \omega_{k+\delta} - \Delta - \frac{\mathrm{i}\kappa_{k+\delta}}{2} \end{pmatrix},$$

which includes three modes $k-\delta, k, k+\delta$ involved in nearest-neighbor scattering (see Fig. 2B). Explicitly,

$$\begin{aligned} S_{21}[\omega] &= \frac{\kappa_{\mathrm{ex},k}}{\left(\frac{\kappa_k}{2} - \mathrm{i}(\omega-\omega_k)\right) + \frac{K^2_{p,p+\delta,k,k-\delta} n_p n_q}{\frac{\kappa_{k-\delta}}{2} - \mathrm{i}(\omega-\omega_{k-\delta}-\Delta)} + \frac{K^2_{p,p+\delta,k,k+\delta} n_p n_q}{\frac{\kappa_{k+\delta}}{2} - \mathrm{i}(\omega-\omega_{k+\delta}+\Delta)}} = \\ &= \frac{\kappa_{\mathrm{ex}}}{\kappa/2 - \mathrm{i}(\omega-\omega_k) + \frac{g^2}{\kappa/2 - \mathrm{i}(\omega-\omega_{k-\delta}-\Delta)} + \frac{g^2}{\kappa/2 - \mathrm{i}(\omega-\omega_{k+\delta}+\Delta)}}, \end{aligned} \tag{S25}$$

where in the last equation we ignored weak linewidth and matrix element dependence on mode number.

By fitting individual avoided crossings on resonance we extract coupling g, f'_k, $f'_{k\pm\delta}$ (note that mode frequencies in Eq. (S25) are different from undriven ones) and linewidth κ (also can be different from undriven case). If experimental conditions are chosen in a way that f'_k, $f'_{k\pm\delta}$ are almost independent on detuning for a certain detuning range, then whole 2D plot can be calculated where the parameters are extracted from two cuts and a good agreement between theory and experiment can be achieved.

Application for cascades

In case of cascaded scattering Eq. (S24) must be used which is trimmed to contain necessary amount of cascades (e.g. for Fig. 3C we set $i_{\mathrm{max}} = 4$). Again, we assume that mode frequencies as well as linewidths stay nearly constant through the pump detuning range used in the experiment. To decrease the number of fitting parameters we introduced a common scaling factor (due to Kerr effect) for mode frequencies which is a single number for all modes participating in cascaded scattering. Here, instead of fitting the coupling g, we kept the matrix element dependence on mode number, see Eq. (S9), and fitted the factor between g and square root of product of mode numbers participating in particular process. This factor corresponds to either fitting of E_{g} or occupation of pumps (due to systematic error in cryostat insertion loss). Thus in total we have 5 fitting parameters: linewidth (κ_{fit}), frequency correction due to Kerr (α_{fit}), occupation of the pumps $n^{\mathrm{pump}}_{\mathrm{fit}} = \sqrt{n_p n_q}$, and magnitude ($S_{\mathrm{fit}}$) and phase offset ($\varphi_{\mathrm{fit}}$) coming from imperfect calibration of the cryostat. Explicitly, our fitting function is:

$$S_{21}[\omega] = \mathrm{i} S_{\mathrm{fit}} \kappa_{\mathrm{ex},k} \left[(\omega \mathbb{I} - \mathbb{A}_{\mathrm{fit}})^{-1} \right]_{i_{\mathrm{max}}+1, i_{\mathrm{max}}+1} e^{\mathrm{i}\varphi_{\mathrm{fit}}}, \tag{S26}$$

with

$$\begin{cases} \mathbb{A}_{\mathrm{fit}}[n,n] = \omega_{k+(n-1-i_{\mathrm{max}})\delta}(1+\alpha_{\mathrm{fit}}) - (n-1-i_{\mathrm{max}})\Delta - \mathrm{i}\frac{\kappa_{\mathrm{fit}}}{2} \\ \mathbb{A}_{\mathrm{fit}}[m,m-1] = \mathbb{A}[m-1,m] = K_{p,p+\delta,k+(m-1-i_{\mathrm{max}})\delta,k+(m-2-i_{\mathrm{max}})\delta} n^{\mathrm{pump}}_{\mathrm{fit}}. \end{cases} \tag{S27}$$

Many-mode drives problem

In this Section we provide the main points of our treatment of the multimode drive problem: the kinetic equation and the self-consistent approach to the linewidth. Although a rigorous description of such problem requires setting a master/Lindblad formalism, for our phenomenological description we assume that the state of the system is described solely by the occupation numbers, $n_k = \langle \hat{a}_k^\dagger \hat{a}_k \rangle$. The equilibration of the driven JJ chain results from the interplay of two processes: the thermal relaxation process, that aims to relax the occupation numbers n_k to thermal equilibrium at the base temperature, and the external driving that aims to increase occupation of pumped modes. In the process of equilibration, the role of the intrinsic scattering between modes from nonlinearity depends on the mode occupation and strength of the driving.

From a single driven mode to many driven and interacting modes

We start with the general form of the kinetic equation of a single plasmonic mode coupled to a background bath with coupling κ_k and whose population is replenished by an external pump-tone:

$$\dot{n}_k = -\kappa_k (n_k + 1) n_k^{\text{th}} + \kappa_k n_k (n_k^{\text{th}} + 1) + \kappa_{\text{ex},k} n_k^{\text{flux}} = -\kappa_k (n_k - n_k^{\text{th}}) + \kappa_{\text{ex},k} n_k^{\text{flux}}. \tag{S28}$$

Here $n_k^{\text{th}} = (e^{\hbar\omega_k/(k_B T)} - 1)^{-1}$ is the thermal occupation of mode k according to Bose-Einstein distribution at temperature T, κ_k is the total coupling rate to the thermal bath (including coupling to both terminals and to the internal environment), and $\kappa_{\text{ex},k}$ is the coupling rate to each of the external terminals. When n_k^{flux} — the number of injected photons per unit frequency and unit time — is zero, this equation yields the expected exponential in time relaxation of n_k to the thermal occupation. Otherwise, the population of the given mode is increased by an amount set by the ratio of $\kappa_{\text{ex},k}$ and κ_k. The nonequilibrium steady state (NESS) is obtained as a stationary point

$$\dot{n}_k = 0 \Rightarrow n_k = n_k^{\text{th}} + \frac{\kappa_{\text{ex},k}}{\kappa_k} n_k^{\text{flux}}, \tag{S29}$$

and is characterized by the excess occupation proportional to the external photon flux.

We generalize Eq. (S28) to the multi-mode case, where all modes interact with each other and multiple modes are pumped. In the experiment, the photon flux is non-zero for the lowest 19 modes, and is related to the noise power spectral density P at the input of the cavity as $n_k^{\text{flux}} = P/(h\omega_k)$. Besides that, the essential missing contribution to the kinetic equation (S28) are inter-mode scattering processes caused by the nonlinearity, that conserve both energy and momentum. These processes are accounted by the collision integral, that depends on the occupation factors of all modes and on the transition probability arising from H_{int}, calculated with a Fermi golden rule approach. For each scattering process involving a set of modes k, p, q_1, q_2, the collision integral consists of an in- and an out-scattering component (*32*):

$$I_{\text{in}}[k] = \sum_p \sum_{q_1 > q_2} W_{q_1 q_2 \to p,k} (1 + n_p)(1 + n_k) n_{q_1} n_{q_2}, \quad I_{\text{out}}[k] = -\sum_p \sum_{q_1 > q_2} W_{p,k \to q_1 q_2} n_p n_k (1 + n_{q_1})(1 + n_{q_2}), \tag{S30}$$

where W depends on the matrix element as ($\hbar = 1$)

$$W_{q_1 q_2 \to p,k} = W_{p,k \to q_1 q_2} = 2\pi |K_{q_1 q_2 p k}|^2 \delta(\omega_{q_1} + \omega_{q_2} - \omega_p - \omega_k). \tag{S31}$$

In the above sums, we exclude by hand the unphysical cases of self-decay, i.e. $q_1 = k,\ q_2 = k$.

Finally, the full kinetic equation including collision integral is written as:

$$\dot{n}_k = -\kappa_k (n_k - n_k^{\mathrm{th}}) + \kappa_{\mathrm{ex},k} n_k^{\mathrm{flux}} + I_{\mathrm{in}}[k] + I_{\mathrm{out}}[k], \tag{S32}$$

where we use a set of modes up to a certain cutoff, $k = 1, \ldots, k_{\mathrm{max}}$.

Self-consistent linewidth and final excess linewidth

The transition probability (S31) contains a delta-function in energy. In practice, every mode has a Lorentzian shape with a finite linewidth. Hence, we replace the δ-function by a broadened form, $\delta(\omega) \to \delta_\gamma(\omega)$,

$$\delta_\gamma(\omega) = \frac{1}{\pi} \frac{\gamma}{\gamma^2 + \omega^2}, \tag{S33}$$

where 2γ physically corresponds to the total linewidth. Since four convolved Lorentzians produce a Lorentzian with linewidth given by the sum of the original linewidths, we replace the δ function in Eq. (S31) by the expression Eq. (S33) above with broadening γ given by:

$$\gamma_{k,p,q_1,q_2} = \sum_{j \in [k,p,q_1,q_2]} (2\kappa_{\mathrm{ex},j} + \kappa_{\mathrm{i},j} + \delta\kappa_j)/2, \tag{S34}$$

which depends symmetrically on the four mode numbers that participate in the scattering process (this is required to fulfill the detailed balance). The first two terms in the sum are originating from external broadening and internal losses. The third contribution to the sum in Eq. (S34), $\delta\kappa_j$, is the (self-consistent) excess linewidth of mode j due to the intrinsic scattering caused by the nonlinearity.

Within the framework of kinetic equation, the excess intrinsic linewidth is obtained from the diagonal part of the linearized collision integral,

$$\delta\kappa_k = \frac{\pi^5 E_{\mathrm{g}}^2}{16 N^6} \sum_{p,q_1,q_2} k p q_1 q_2\, \delta(\omega_k + \omega_p - \omega_{q_1} - \omega_{q_2}) [n_p(1 + n_{q_1} + n_{q_2}) - n_{q_1} n_{q_2}] \sum_{s_1,s_2,s_3=\pm} \delta_{k+s_1 p+s_2 q_1+s_3 q_2,0}, \tag{S35}$$

where again the first sum excludes self-decays, and we used explicit expression for the matrix element from Eq. (S9).

Obtaining NESS and linewidth numerically

In order to model the increase of the linewidth in observed in the experimental data, we calculate the NESS of the kinetic equation with fixed $n_k^{\rm flux}$. In the simulations we used $k_{\rm max} = 173$ and smaller values $k_{\rm max} = 100$ to make sure that occupation of modes with numbers much below $k_{\rm max}$ are insensitive to precise value of the $k_{\rm max}$. We get the NESS by time evolving the distribution function according to Eq. (S32) until the norm of the change of n_k between two consecutive time steps becomes smaller than tolerance, $\sum_k |\delta n_k|^2 \leq 10^{-14}$. During the time evolution with time step $\Delta t = 0.01/\kappa_0$ ($\kappa_0 = 1.7$ MHz is an average value for κ_k), we update the value of $\delta\kappa_j$ using Eq. (S35) at every $10^{\rm th}$ time step. After the convergence is reached, we use the converged values of the distribution function in the NESS, n_k, to calculate $\delta\kappa_j$ that is plotted in Fig. 5C.

Although the system is fully described by the three energy parameters, achieving agreement between simulations and experiments required introducing two *additional* phenomenological fitting parameters, resulting in the modified kinetic equation:

$$\dot{n}_k = -(\kappa_k + \delta\kappa_{{\rm i},k})(n_k - n_k^{\rm th}) + \alpha\kappa_{{\rm ex},k} n_k^{\rm flux} + I_{\rm in}[k] + I_{\rm out}[k]. \tag{S36}$$

In this equation the following parameters are introduced:

1. The first parameter is a frequency-independent correction to the insertion loss of the fridge, α, which affects the magnitude of the term proportional to $n_k^{\rm flux}$. We set $\alpha = 5$ based on comparisons between the 20 dB data and simulations. As point of comparison, the measured nonlinearity in $K/2\pi = 16$ kHz/ph from Fig. 2E, indicates an insertion-loss correction of 3. The insertion-loss correction determined from nonlinearities thus indicates that our independent throughline insertion loss measurements underestimate the total insertion loss by $5 - 7$ dB.

2. For stronger drives, we introduce an additional internal loss, $\delta\kappa_{{\rm i},k}$, for the driven modes with numbers $k = 1, \ldots, 19$, Setting $\delta\kappa_{{\rm i},k} = 6$ MHz for the 5 dB data results in a good match between theory and experiment. The introduction of $\delta\kappa_{{\rm i},k}$ is justified by the evident presence of additional losses in the lowest measured modes at strong drives (e.g., see the 0 dB data in Fig. 5C). This excess loss might be attributed to phase slips, which are beyond the scope of our theoretical model.

Estimate of the number of decay channels

Lastly, we comment on the number of decay channels arising in the multimode drive case for a mode that is not being driven. As an example, we consider mode $k = 80$. In order to visualize the number of decay channels we first obtain numerically NESS. After this, we plot the contribution of individual terms in the Eq. (S35) to the linewidth of the k-th mode. The decay processes of mode k are labeled by three numbers, $k, p \to q_1, q_2$, however the number p is deduced from momentum conservation, leaving only two independent mode numbers, q_1 and q_2. In order to avoid dealing with multiple signs of momenta ($s_{1,2,3} = \pm 1$ in Eq. (S35)), we plot the contribution to $\delta\kappa_k$ as a function of $s_2 q_1$ and $s_3 q_2$, so that they take both positive and negative values now. The

total excess linewidth results from the sum of all matrix elements, but here we want to get an insight into the mostly contributing decay channels.

First we show the matrix plot in Fig. S3A where we mark by black color the values of (s_2q_1, s_3q_2) that exhaust the 95% of the total linewidth. From here we see that relevant processes are of two types. The first is scattering to nearby modes (i.e. when either q_1 or q_2 is in the vicinity of $k = 80$), which corresponds to the up-right and bottom-left features in the matrix plot. This scattering is shown schematically in Fig. 5A of the main text. The second scattering process is reminiscent of the scattering process discussed in (*43*) for an infinite Josephson junction chain at thermal equilibrium, where k relaxes by scattering off a plasmon with much smaller mode number p and negative momentum. In our matrix plot, this corresponds to the black entries following a diagonal trend in Fig. S3A.

In addition, in Fig. S3B we show the relative cumulative rate, obtained by progressively summing contributions of all decay channels, from the largest to the smallest one, normalized by total decay rate. This can be used to visualize the *number* of decay channels for mode k. Once the number is divided by two, to account for the indistinguishability of q_1 and q_2, we obtain an estimate of a bit more than 100 channels to achieve a cumulative proportion of 95%.

Estimation of scattered photons

The noise data in Fig. 4B (see also Fig. S8 for a different pumping configuration) can be further analyzed quantitatively. For that it is necessary to calibrate the measurement chain and determine the amplifier-referred gain, G, and added noise (see Fig. S10). The background in Fig. 4B and Fig. S8 is set by the added noise within a 270 Hz band, which was measured to be approximately -103 dBm for the frequency range used in those measurements. Regions where P_N significantly exceeds the background can be interpreted as cavity emission at a rate proportional to κ_ex. Using an independent calibration of the input-referred gain, G, the number of photons inside the mode can be estimated as $\frac{P_\mathrm{N}}{G\kappa_\mathrm{ex}}\frac{1}{hf_\mathrm{N}}$. For $P_\mathrm{N} = -75$ dBm, this estimate yields approximately half a photon for a typical $\kappa_\mathrm{ex} = 2\pi \times 0.4$ MHz, which agrees well with the estimation for resonant scattering $0.5 - 1$, given by $n_k \cdot g/\kappa$.

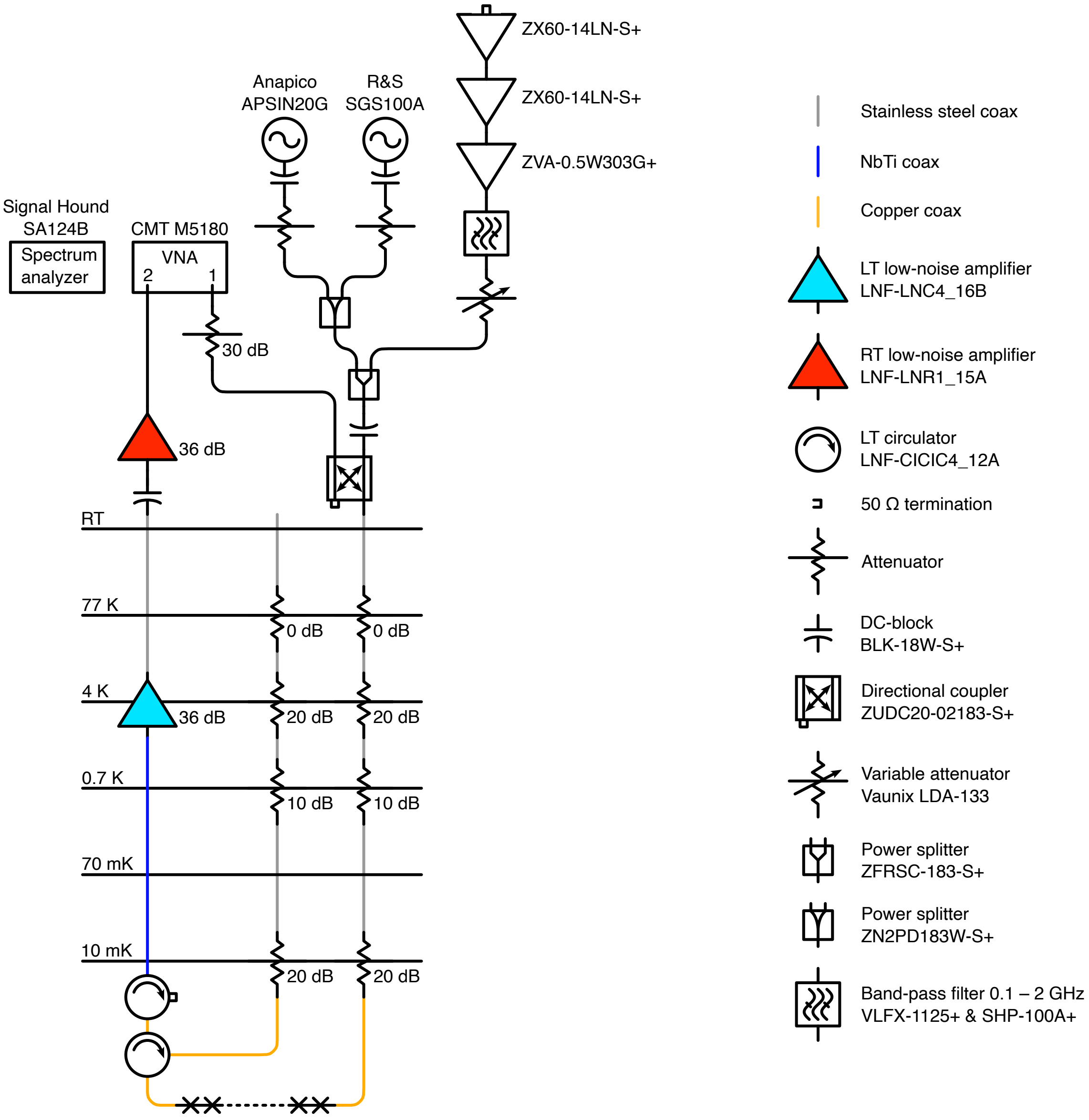

Figure S1: **Experimental setup used in this work.** Power splitters and directional coupler combines microwave radiation from all sources and feeds it into the fridge. Passed though the series of cold attenuators and the device the signal is amplified by a cascade of low-temperature and room-temperature amplifiers and is read-out by vector network analyzer (VNA). Alternatively, the signal out-coming from the fridge can be detected by the spectrum analyzer. A cascade of RT amplifiers plays role of a wide band noise source (the band is defined by a series of low-pass and high-pass filters) where the variable attenuator allows us to control the noise temperature at the input of cryostat roughly in a range of $300 - 3 \cdot 10^8$ K.

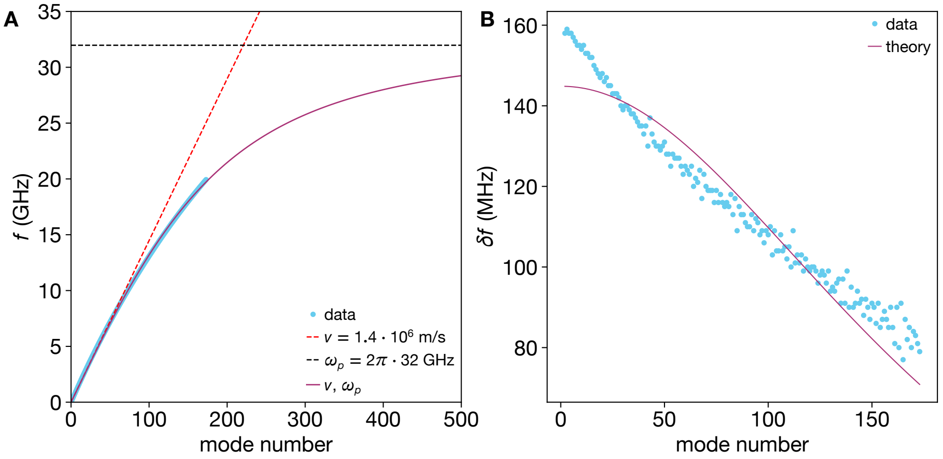

Figure S2: **Dispersion curve of the JJ-chain. (A)** Blue data points represent the frequencies of the first 173 modes measured using the two-tone spectroscopy technique (see details in the text). The blue solid line corresponds to a fit to Eq. (S1), while the two dashed lines indicate two relevant quantities: the speed of light, v, and the plasma frequency of a single junction, ω_p. **(B)** Mode spacing. Blue data points represent experimental data (the same as in **(A)**). The theoretical curve is obtained by differentiating the fit from panel **(A)**.

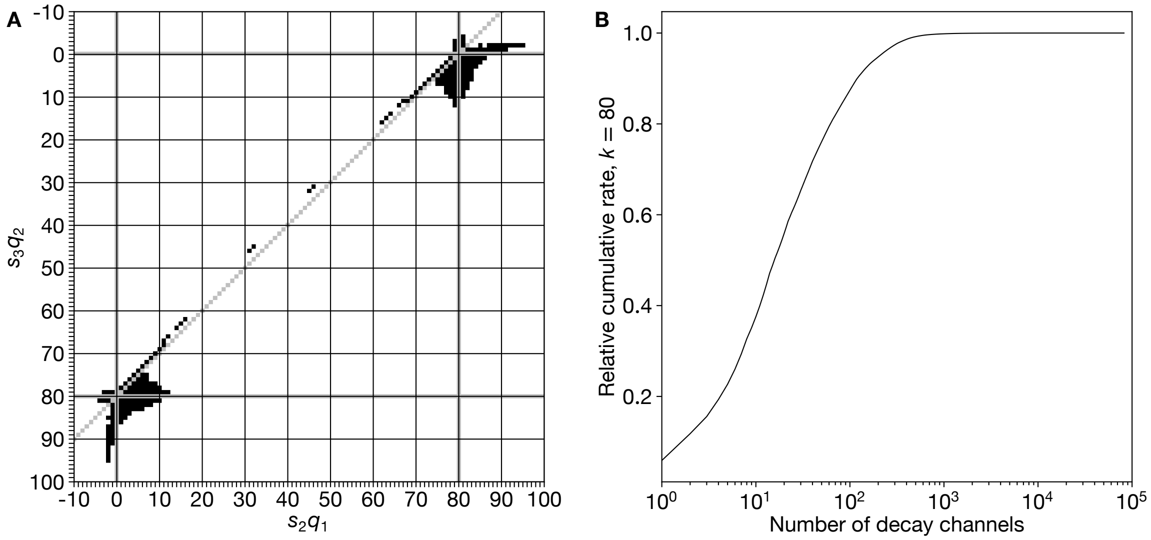

Figure S3: **Analysis of decay channels for a not driven mode. A.** Matrix plot of contributions to the decay rate of mode $k = 80$. Each entry corresponds to a different decay channel, identified by s_2q_1 and s_3q_2 in Eq. (S35). Gray lines indicate the unphysical cases of self-decay process (either q_1 or q_2 being equal to k), or mode numbers q_1, q_2, $p = 0$, which is just a numerical artifice. **B.** Relative cumulative rate for the same mode $k = 80$, which allows to easily estimate the number of decay channels needed to accumulate a given percentage of the total rate.

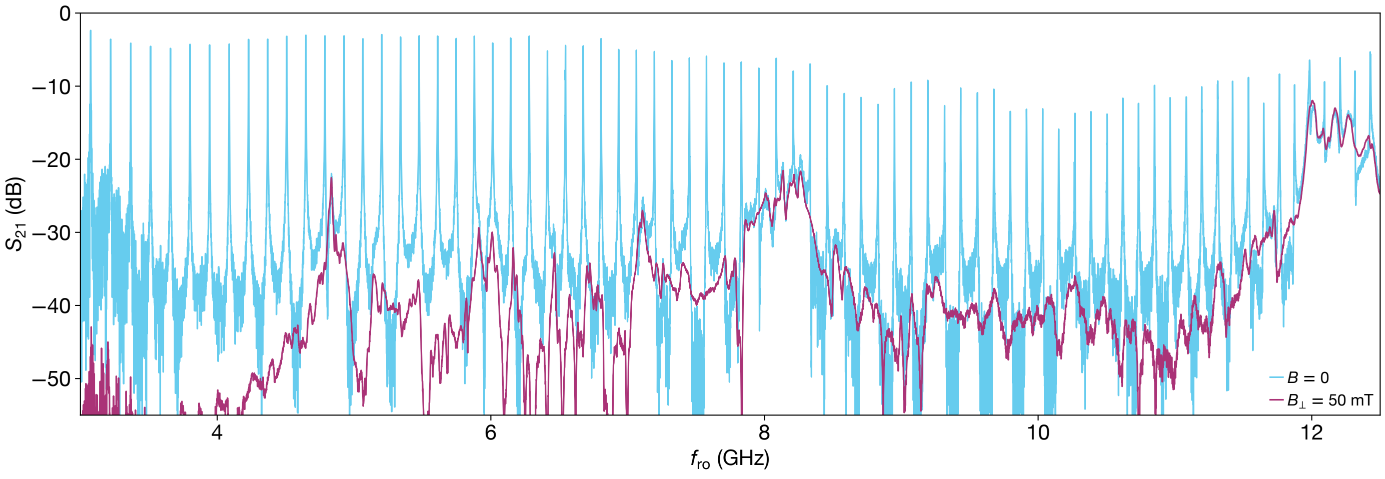

Figure S4: **Single tone data without background subtraction.** Single-tone transmission data, S_{21}, is presented at zero magnetic field and at $B_\perp = 50$ mT, where the JJ-chain transitions to normal state. The normal state data serves as a background representing cross-talk between the two ports of the printed circuit board, which can be subtracted (in linear voltage ratio units). In Fig. 1B of the main text, the S_{21} data was subtracted for visualization purposes, whereas in the remaining figures, the unsubtracted data is reported. Data within frequency ranges exhibiting high cross-talk (e.g., at $f_{ro} \sim 8$ GHz) were compared with the $B_\perp = 50$ mT trace and excluded from the analysis if the subtraction procedure caused significant changes in the fitted resonance parameters (e.g., low attenuation data in Fig. 5C for $k \approx 60$).

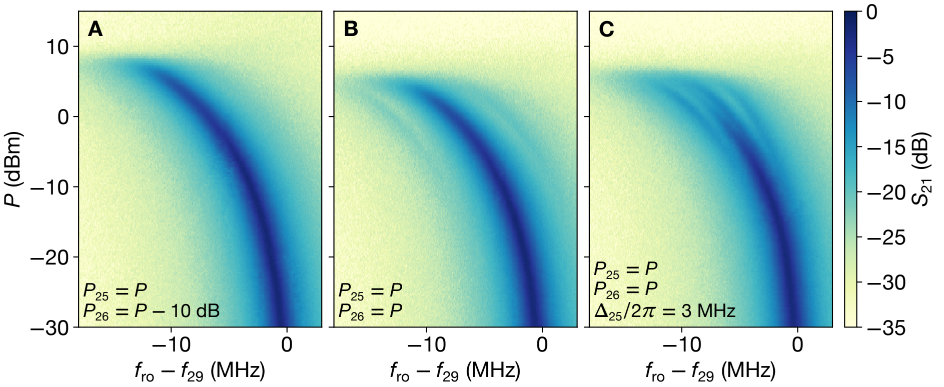

Figure S5: **Pump imbalance and pump detuning effects.** Three-tone spectroscopy data for the similar configuration of pumps ($p = 25$, $q = 26$) and read-out ($k = 29$) as in Fig. 1E. **A.** Pump powers are imbalanced, with P_{25} higher than P_{26} by 10 dB. **B.** Pump powers are equal, $P_{25} = P_{26} = P$. **C.** Pump powers are equal, but the pump frequency of mode 25 is blue-detuned by 3 MHz compared to panels (**A, B**). This is the same data as presented in Fig. 1E.

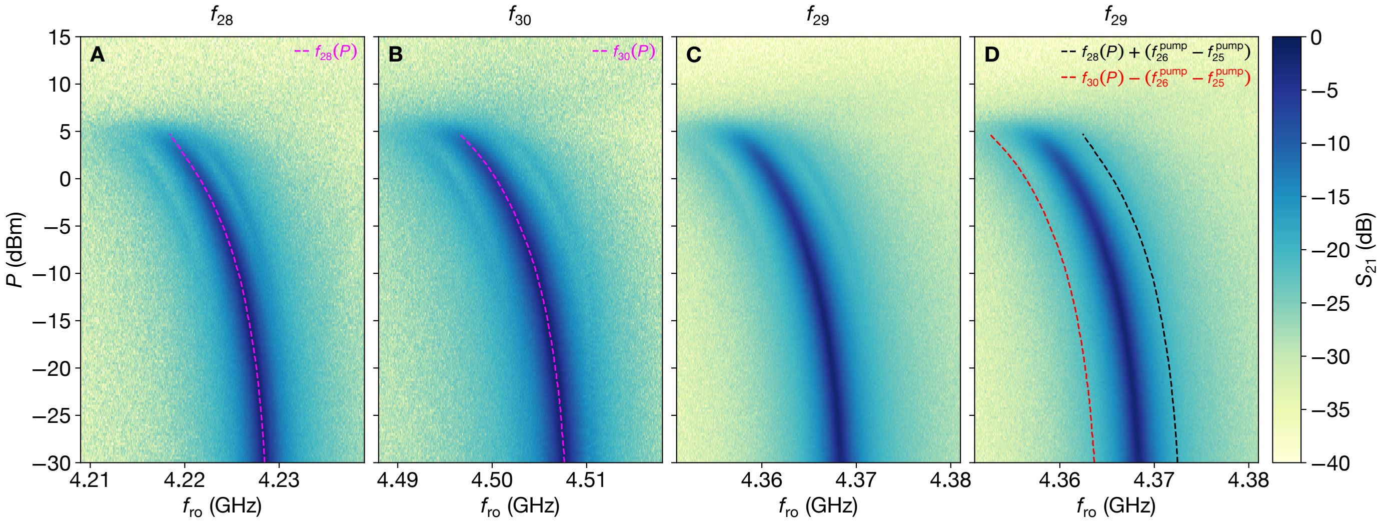

Figure S6: **Sideband feature energetics** Three-tone spectroscopy data for the same configuration of pumps ($p = 25$, $q = 26$) as in Fig. S5B. In all panels, the pumping configuration was kept identical, and only the read-out tone (k) was varied. **A-B.** S_{21} measurements around modes 28 and 30. The resonances $f_{28}(P)$ and $f_{30}(P)$ were determined as functions of the pump power P. **C-D.** The same data as in Fig. S5B. In panel **D**, two guides are overlaid, showing predictions for a four-wave mixing-like interaction based on a quasi-classical energy conservation argument.

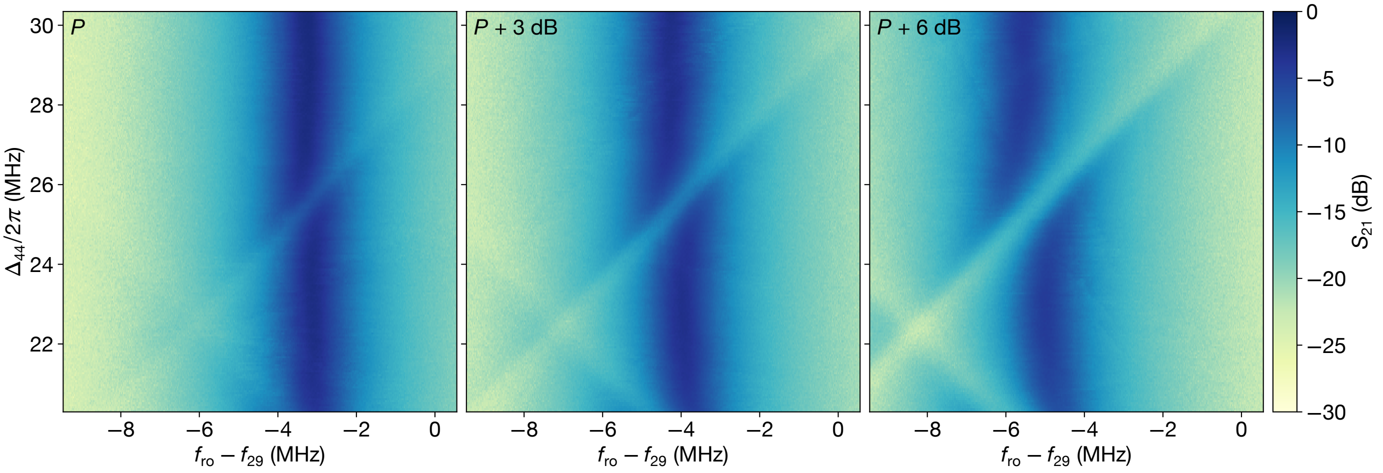

Figure S7: **Avoided crossing power dependence** S_{21} data near the avoided crossing for a similar pumping configuration as in Fig. 2B. A zoomed-in view of the top avoided crossing is shown in each panel. The pump power for both pump tones was varied simultaneously, increasing by 3 dB in each panel. Data in Fig. 2D correspond to cuts at the avoided crossing from this dataset.

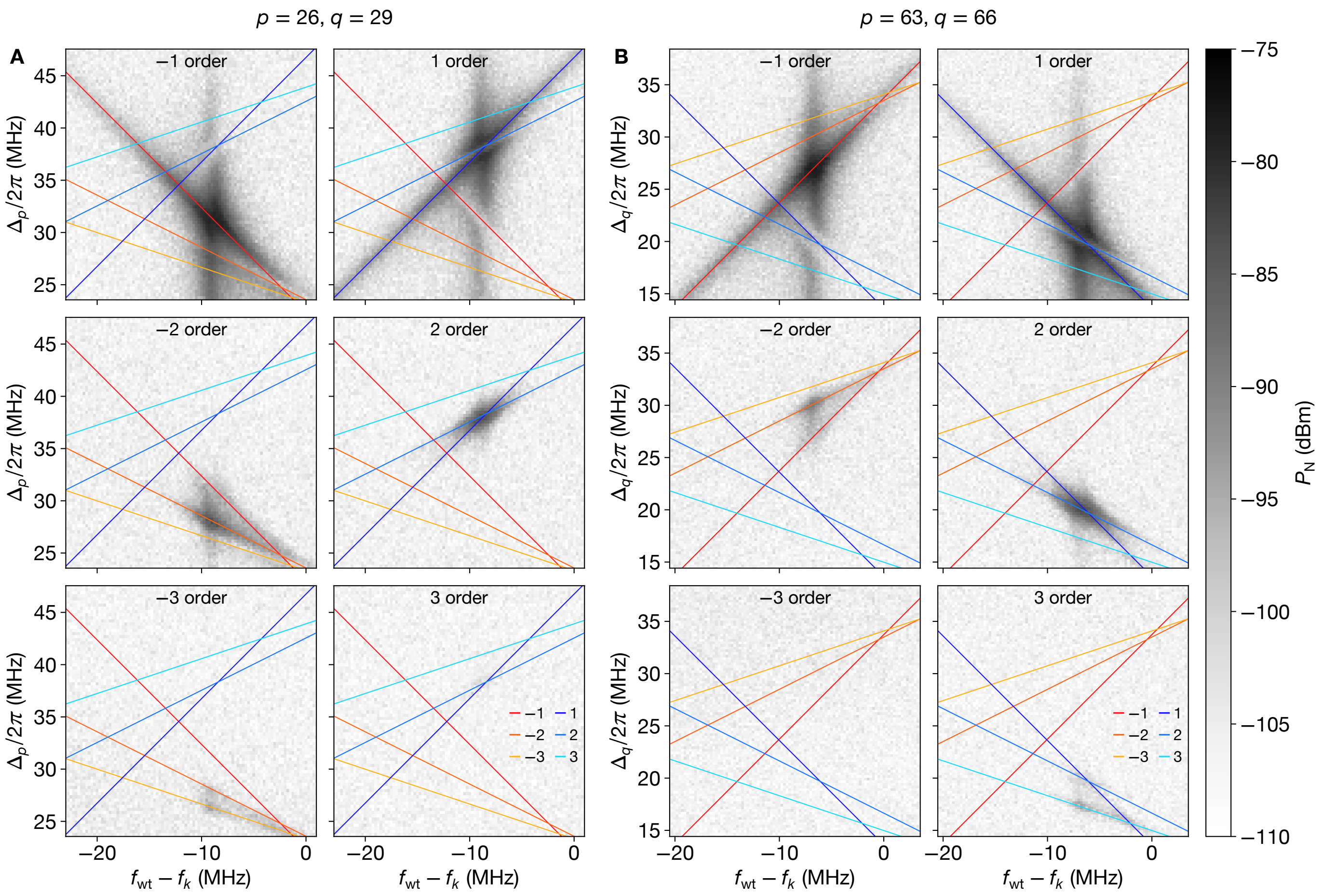

Figure S8: **Observation of scattered photons via noise measurements** Noise measurement data for a different pumping configuration with $p, q < k$. In **B**, the data from Fig. 4B is replotted. A weak tone is applied at $k = 46$ in both panels, and the pump powers for **A** were chosen to result in a similar matrix element as the pumping configuration in **B**.

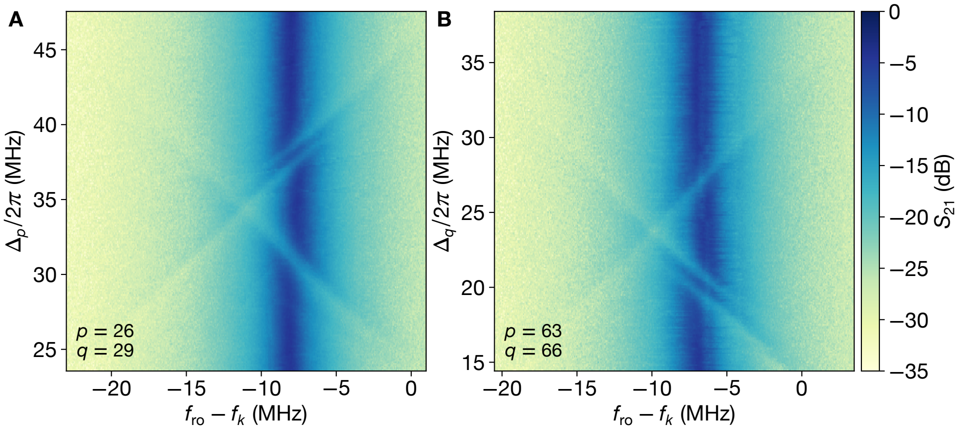

Figure S9: **Moderate pump power three-tone spectroscopy data A.** Transmission data measured under the same pumping configuration as the noise data in Fig. S8A. **B.** Transmission data measured under the same pumping configuration as the noise data in Fig. 4B and Fig. S8B.

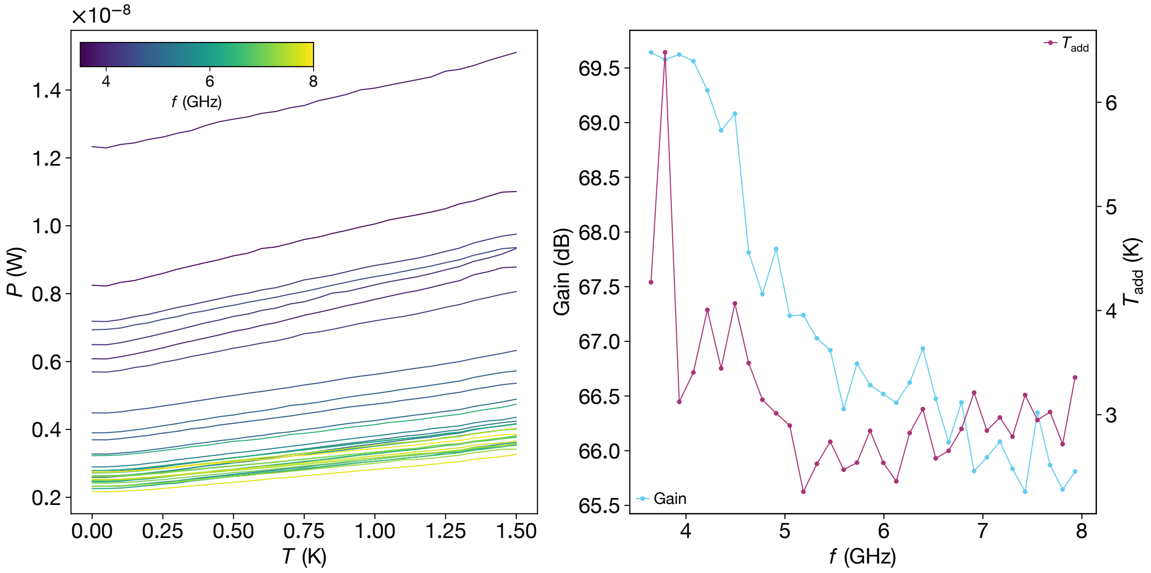

Figure S10: **Calibration of measurement chain of the setup** The power measured by a spectrum analyzer, P, emitted from the cryostat, is shown for different center frequencies (denoted by color) as a function of the mixing chamber plate temperature, T. All data were recorded in 15 MHz bands centered at the same frequencies as the data in Fig. 5D-E, to calibrate the input-referred gain, G, and the added noise temperature, T_{add}. The gain and added noise temperature were extracted from a linear fit of P versus T using the relation $P = G(T + T_{\text{add}})$ for $0.25 \leq T \leq 1.4$ K.

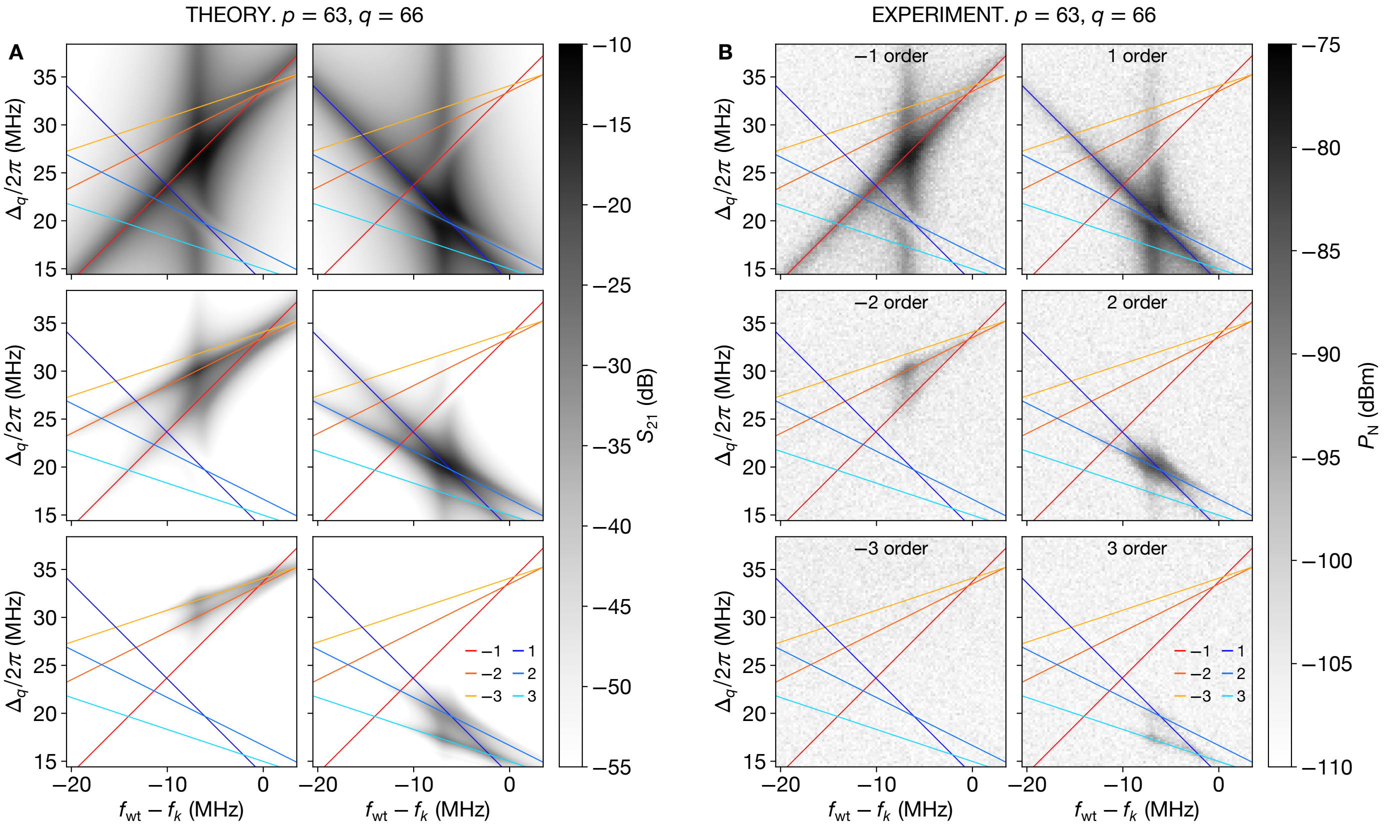

Figure S11: **Comparison of theory and experiment for cascades A.** Theoretical calculation of off-diagonal scattering parameter $S_{21}[k \to k + i\delta]$ between mode k and $k + i\delta$ using Eq. (S26). The panel is plotted using matrix element fitted from Fig. S9B which is measured at the same pumping configuration as panel **B**. In **B**, the data from Fig. 4B is replotted.

Table S1: **Estimation of JJ-chain parameters.** The data in column 'Value' are obtained from dispersion fit and geometry of the transmission line. In 'Consistency check' the data are calculated from dispersion fit and *assuming* 50 – 100 fF/μm^2 of specific capacitance (*67, 68*). For each column the theoretically calculated parameter is marked with an asterisk.

Parameter	Value	Consistency check
E_g/h	3.9 THz*	2.9 – 5.8 THz
E_J/h	47 GHz	32 – 64 GHz
E_C/h	11 GHz	8 – 16 GHz*
Z	13 kΩ	10 – 20 kΩ

REFERENCES

1. R. Peierls, Zur kinetischen Theorie der Wärmeleitung in Kristallen. *Ann. Phys.* **395**, 1055–1101 (1929).

2. E. Fermi, J. R. Pasta, S. M. Ulam, "Studies of nonlinear problems" (Tech. Rep., Los Alamos National Laboratory,1955).

3. S. Nazarenko, *Wave Turbulence* (Springer, 2011); 10.1007/978-3-642-15942-8.

4. H. Xu, D. Mason, L. Jiang, J. G. E. Harris, Topological energy transfer in an optomechanical system with exceptional points. *Nature* **537**, 80–83 (2016).

5. Y. S. S. Patil, J. Höller, P. A. Henry, C. Guria, Y. Zhang, L. Jiang, N. Kralj, N. Read, J. G. E. Harris, Measuring the knot of non-Hermitian degeneracies and non-commuting braids. *Nature* **607**, 271–275 (2022).

6. F. Ruesink, J. P. Mathew, M.-A. Miri, A. Alù, E. Verhagen, Optical circulation in a multimode optomechanical resonator. *Nat. Commun.* **9**, 1798 (2018).

7. V. V. Sivak, S. Shankar, G. Liu, J. Aumentado, M. H. Devoret, Josephson array-mode parametric amplifier. *Phys. Rev. Appl.* **13**, 024014 (2020).

8. R. Naik, N. Leung, S. Chakram, P. Groszkowski, Y. Lu, N. Earnest, D. C. McKay, J. Koch, D. I. Schuster, Random access quantum information processors using multimode circuit quantum electrodynamics. *Nat. Commun.* **8**, 1904 (2017).

9. C. T. Hann, C.-L. Zou, Y. Zhang, Y. Chu, R. J. Schoelkopf, S. M. Girvin, L. Jiang, Hardware-efficient quantum random access memory with hybrid quantum acoustic systems. *Phys. Rev. Lett.* **123**, 250501 (2019).

10. A. R. Matanin, K. I. Gerasimov, E. S. Moiseev, N. S. Smirnov, A. I. Ivanov, E. I. Malevannaya, V. I. Polozov, E. V. Zikiy, A. A. Samoilov, I. A. Rodionov, S. A. Moiseev, Toward highly efficient multimode superconducting quantum memory. *Phys. Rev. Appl.* **19**, 034011 (2023).

11. J. P. Pekola, B. Karimi, Quantum thermalization via multiwave mixing. *Phys. Rev. Res.* **6**, L042023 (2024).

12. N. M. Sundaresan, Y. Liu, D. Sadri, L. J. Szőcs, D. L. Underwood, M. Malekakhlagh, H. E. Türeci, A. A. Houck, Beyond strong coupling in a multimode cavity. *Phys. Rev. X* **5**, 021035 (2015).

13. B. A. Moores, L. R. Sletten, J. J. Viennot, K. W. Lehnert, Cavity quantum acoustic device in the multimode strong coupling regime. *Phys. Rev. Lett.* **120**, 227701 (2018).

14. Y. Guo, R. M. Kroeze, V. D. Vaidya, J. Keeling, B. L. Lev, Sign-changing photon-mediated atom interactions in multimode cavity quantum electrodynamics. *Phys. Rev. Lett.* **122**, 193601 (2019).

15. D. B. Haviland, P. Delsing, Cooper-pair charge solitons: The electrodynamics of localized charge in a superconductor. *Phys. Rev. B* **54**, R6857–R6860 (1996).

16. E. Chow, P. Delsing, D. B. Haviland, Length-scale dependence of the superconductor-to-insulator quantum phase transition in one dimension. *Phys. Rev. Lett.* **81**, 204–207 (1998).

17. I. M. Pop, I. Protopopov, F. Lecocq, Z. Peng, B. Pannetier, O. Buisson, W. Guichard, Measurement of the effect of quantum phase slips in a Josephson junction chain. *Nat. Phys.* **6**, 589–592 (2010).

18. A. Ergül, J. Lidmar, J. Johansson, Y. Azizoğlu, D. Schaeffer, D. B. Haviland, Localizing quantum phase slips in one-dimensional Josephson junction chains. *New J. Phys.* **15**, 095014 (2013).

19. R. M. Bradley, S. Doniach, Quantum fluctuations in chains of Josephson junctions. *Phys. Rev. B* **30**, 1138–1147 (1984).

20. M.-S. Choi, J. Yi, M. Y. Choi, J. Choi, S.-I. Lee, Quantum phase transitions in Josephson-junction chains. *Phys. Rev. B* **57**, R716–R719 (1998).

21. R. Fazio, H. van der Zant, Quantum phase transitions and vortex dynamics in superconducting networks. *Phys. Rep.* **355**, 235–334 (2001).

22. B. Yurke, M. L. Roukes, R. Movshovich, A. N. Pargellis, A low-noise series-array Josephson junction parametric amplifier. *Appl. Phys. Lett.* **69**, 3078–3080 (1996).

23. M. A. Castellanos-Beltran, K. D. Irwin, G. C. Hilton, L. R. Vale, K. W. Lehnert, Amplification and squeezing of quantum noise with a tunable Josephson metamaterial. *Nat. Phys.* **4**, 929–931 (2008).

24. N. Bergeal, F. Schackert, M. Metcalfe, R. Vijay, V. E. Manucharyan, L. Frunzio, D. E. Prober, R. J. Schoelkopf, S. M. Girvin, M. H. Devoret, Phase-preserving amplification near the quantum limit with a Josephson ring modulator. *Nature* **465**, 64–68 (2010).

25. V. E. Manucharyan, J. Koch, L. I. Glazman, M. H. Devoret, Fluxonium: Single cooper-pair circuit free of charge offsets. *Science* **326**, 113–116 (2009).

26. N. A. Masluk, I. M. Pop, A. Kamal, Z. K. Minev, M. H. Devoret, Microwave characterization of Josephson junction arrays: Implementing a low loss superinductance. *Phys. Rev. Lett.* **109**, 137002 (2012).

27. R. Kuzmin, R. Mencia, N. Grabon, N. Mehta, Y.H. Lin, V. E. Manucharyan, Quantum electrodynamics of a superconductor–insulator phase transition. *Nat. Phys.* **15**, 930–934 (2019).

28. S. Mukhopadhyay, J. Senior, J. Saez-Mollejo, D. Puglia, M. Zemlicka, J. M. Fink, A. P. Higginbotham, Superconductivity from a melted insulator in Josephson junction arrays. *Nat. Phys.* **19**, 1630–1635 (2023).

29. S. Léger, J. Puertas-Martínez, K. Bharadwaj, R. Dassonneville, J. Delaforce, F. Foroughi, V. Milchakov, L. Planat, O. Buisson, C. Naud, W. Hasch-Guichard, S. Florens, I. Snyman, N. Roch, Observation of quantum many-body effects due to zero point fluctuations in superconducting circuits. *Nat. Commun.* **10**, 5259 (2019).

30. N. Mehta, R. Kuzmin, C. Ciuti, V. E. Manucharyan, Down-conversion of a single photon as a probe of many-body localization. *Nature* **613**, 650–655 (2023).

31. N. Crescini, S. Cailleaux, W. Guichard, C. Naud, O. Buisson, K. W. Murch, N. Roch, Evidence of dual Shapiro steps in a Josephson junctions array. *Nat. Phys.* **19**, 851–855 (2023).

32. J. Lin, K. A. Matveev, M. Pustilnik, Thermalization of acoustic excitations in a strongly interacting one-dimensional quantum liquid. *Phys. Rev. Lett.* **110**, 016401 (2013).

33. I. Carusotto, C. Ciuti, Quantum fluids of light. *Rev. Mod. Phys.* **85**, 299–366 (2013).

34. C. Eichler, Y. Salathe, J. Mlynek, S. Schmidt, A. Wallraff, Quantum-limited amplification and entanglement in coupled nonlinear resonators. *Phys. Rev. Lett.* **113**, 110502 (2014).

35. S. W. Jolin, G. Andersson, J. C. R. Hernández, I. Strandberg, F. Quijandría, J. Aumentado, R. Borgani, M. O. Tholén, D. B. Haviland, Multipartite entanglement in a microwave frequency comb. *Phys. Rev. Lett.* **130**, 120601 (2023).

36. E. Zakka-Bajjani, F. Nguyen, M. Lee, L. R. Vale, R. W. Simmonds, J. Aumentado, Quantum superposition of a single microwave photon in two different 'colour' states. *Nat. Phys.* **7**, 599–603 (2011).

37. F. D. M. Haldane, 'Luttinger liquid theory' of one-dimensional quantum fluids. I. Properties of the Luttinger model and their extension to the general 1D interacting spinless Fermi gas. *J. Phys. C. Solid State Phys.* **14**, 2585–2609 (1981).

38. A. Imambekov, T. L. Schmidt, L. I. Glazman, One-dimensional quantum liquids: Beyond the Luttinger liquid paradigm. *Rev. Mod. Phys.* **84**, 1253–1306 (2012).

39. V. V. Deshpande, M. Bockrath, L. I. Glazman, A. Yacoby, Electron liquids and solids in one dimension. *Nature* **464**, 209–216 (2010).

40. G. Barak, H. Steinberg, L. N. Pfeiffer, K. W. West, L. Glazman, F. von Oppen, A. Yacoby, Interacting electrons in one dimension beyond the Luttinger-liquid limit. *Nat. Phys.* **6**, 489–493 (2010).

41. S. Wang, S. Zhao, Z. Shi, F. Wu, Z. Zhao, L. Jiang, K. Watanabe, T. Taniguchi, A. Zettl, C. Zhou, F. Wang, Nonlinear Luttinger liquid plasmons in semiconducting single-walled carbon nanotubes. *Nat. Mater.* **19**, 986–991 (2020).

42. M. F. Gely, A. Sanz Mora, S. Yanai, R. van der Spek, D. Bothner, G. A. Steele, Apparent nonlinear damping triggered by quantum fluctuations. *Nat. Commun.* **14**, 7566 (2023).

43. M. Bard, I. V. Protopopov, A. D. Mirlin, Decay of plasmonic waves in Josephson junction chains. *Phys. Rev. B* **98**, 224513 (2018).

44. Y. Krupko, V. D. Nguyen, T. Weißl, É. Dumur, J. Puertas, R. Dassonneville, C. Naud, F. W. J. Hekking, D. M. Basko, O. Buisson, N. Roch, W. Hasch-Guichard, Kerr nonlinearity in a superconducting Josephson metamaterial. *Phys. Rev. B* **98**, 094516 (2018).

45. Y. Krupko, V. D. Nguyen, T. Weißl, É. Dumur, J. Puertas, R. Dassonneville, C. Naud, F. W. J. Hekking, D. M. Basko, Kerr nonlinearity in a superconducting Josephson metamaterial. *Phys. Rev. B* **108**, 219904 (2023).

46. T. Weißl, B. Küng, E. Dumur, A. K. Feofanov, I. Matei, C. Naud, O. Buisson, F. W. J. Hekking, W. Guichard, Kerr coefficients of plasma resonances in Josephson junction chains. *Phys. Rev. B* **92**, 104508 (2015).

47. C. Eichler, A. Wallraff, Controlling the dynamic range of a Josephson parametric amplifier. *EPJ Quantum Technol.* **1**, 2 (2014).

48. P. R. Muppalla, O. Gargiulo, S. I. Mirzaei, B. P. Venkatesh, M. L. Juan, L. Grünhaupt, I. M. Pop, G. Kirchmair, Bistability in a mesoscopic Josephson junction array resonator. *Phys. Rev. B* **97**, 024518 (2018).

49. C. K. Andersen, A. Kamal, N. A. Masluk, I. M. Pop, A. Blais, M. H. Devoret, Quantum versus classical switching dynamics of driven dissipative Kerr resonators. *Phys. Rev. Appl.* **13**, 044017 (2020).

50. M. Aspelmeyer, T. J. Kippenberg, F. Marquardt, Cavity optomechanics. *Rev. Mod. Phys.* **86**, 1391–1452 (2014).

51. M. Houzet, L. I. Glazman, Microwave spectroscopy of a weakly pinned charge density wave in a superinductor. *Phys. Rev. Lett.* **122**, 237701 (2019).

52. A. Burshtein, M. Goldstein, Inelastic decay from integrability. *Quantum* **5**, 020323 (2024).

53. A. Vrajitoarea, R. Belyansky, R. Lundgren, S. Whitsitt, A. V. Gorshkov, A. A. Houck, Ultrastrong light-matter interaction in a multimode photonic crystal. arXiv:2209.14972 [quant-ph] (2022).

54. D. Fraudet, I. Snyman, D. M. Basko, S. Léger, T. Sépulcre, A. Ranadive, G. L. Gal, A. Torras-Coloma, W. Guichard, Direct detection of down-converted photons spontaneously produced at a single Josephson junction. *Phys. Rev. Lett.* **134**, 013804 (2025).

55. B. M. Brubaker, L. Zhong, Y. V. Gurevich, S. B. Cahn, S. K. Lamoreaux, M. Simanovskaia, J. R. Root, S. M. Lewis, S. Al Kenany, K. M. Backes, I. Urdinaran, N. M. Rapidis, T. M. Shokair, K. A. van Bibber, D. A. Palken, M. Malnou, W. F. Kindel, M. A. Anil, K. W. Lehnert, G. Carosi, First results from a microwave cavity axion search at 24 μeV. *Phys. Rev. Lett.* **118**, 061302 (2017).

56. K. W. Léonard, A. V. Bubis, M. Mikalsen, W. F. Schiela, B. H. Elfeky, W. M. Strickland, D. Phan, J. Shabani, A. P. Higginbotham, Microwave radiation at criticality in a hybrid Josephson array. arXiv:2409.09835 [cond-mat.mes-hall] (2024).

57. A. Lingenfelter, D. Roberts, A. A. Clerk, Unconditional Fock state generation using arbitrarily weak photonic nonlinearities. *Sci. Adv.* **7**, eabj1916 (2021).

58. A. McDonald, A. A. Clerk, Exponentially-enhanced quantum sensing with non-Hermitian lattice dynamics. *Nat. Commun.* **11**, 5382 (2020).

59. X. M. de Wit, M. Fruchart, T. Khain, F. Toschi, V. Vitelli, Pattern formation by turbulent cascades. *Nature* **627**, 515–521 (2024).

60. M. T. Bell, B. Douçot, M. E. Gershenson, L. B. Ioffe, A. Petković, Josephson ladders as a model system for 1D quantum phase transitions. *CR Physique* **19**, 484–497 (2018).

61. C. Chamon, D. Green, A. J. Kerman, Superconducting circuit realization of combinatorial gauge symmetry. *PRX Quantum* **2**, 030341 (2021).

62. P. Chandra, L. B. Ioffe, D. Sherrington, Possible glassiness in a periodic long-range Josephson array. *Phys. Rev. Lett.* **75**, 713–716 (1995).

63. A. Blais, A. L. Grimsmo, S. M. Girvin, A. Wallraff, Circuit quantum electrodynamics. *Rev. Mod. Phys.* **93**, 025005 (2021).

64. D. V. Negrov, R. V. Kirtaev, I. V. Kiseleva, E. V. Kondratyuk, A. V. Shadrin, A. V. Zenkevich, O. M. Orlov, E. S. Gornev, G. Y. Krasnikov, Integration of functional elements of resistive nonvolative memory with 1T-1R topology. *Russ. Microelectron.* **45**, 383–395 (2016).

65. S. Gevorgian, L. Linner, E. Kollberg, CAD models for shielded multilayered CPW. *IEEE Trans. Microw. Theory Tech.* **43**, 772–779 (1995).

66. R. N. Simons, *Coplanar Waveguide Circuits, Components, and Systems*, Wiley Series in Microwave and Optical Engineering (John Wiley & Sons, Inc., 2001); 10.1002/0471224758.

67. A. Kerr, S.-K. Pan, A. Lichtenberger, D. Lea, Progress on tunerless SIS mixers for the 200–300 GHz band. *IEEE Microw. Guid. Wave Lett.* **2**, 454–456 (1992).

68. F. Deppe, S. Saito, H. Tanaka, H. Takayanagi, Determination of the capacitance of nm scale Josephson junctions. *J. Appl. Phys.* **95**, 2607–2613 (2004).
